# Parental sex-dependent effects of either maternal or paternal eNOS deficiency on the offspring’s phenotype without transmission of the parental eNOS deficiency to the offspring

**DOI:** 10.3389/fphys.2023.1306178

**Published:** 2023-12-19

**Authors:** Xiaoli Zhang, Christoph Reichetzeder, Yvonne Liu, Johann-Georg Hocher, Ahmed A. Hasan, Ge Lin, Burkhard Kleuser, Liang Hu, Berthold Hocher

**Affiliations:** ^1^ Institute of Pharmacy, Freie Universität Berlin, Berlin, Germany; ^2^ Fifth Department of Medicine (Nephrology/Endocrinology/Rheumatology), University Medical Centre Mannheim, University of Heidelberg, Heidelberg, Germany; ^3^ Faculty of Medicine, HMU–Health and Medical University, Potsdam, Germany; ^4^ Medical Faculty of Charité Universitätsmedizin Berlin, Berlin, Germany; ^5^ Second Medical Faculty, Charles University Prague, Prague, Czechia; ^6^ Reproductive and Genetic Hospital of CITIC-Xiangya, Changsha, China; ^7^ Institute of Reproductive and Stem Cell Engineering, NHC Key Laboratory of Human Stem Cell and Reproductive Engineering, School of Basic Medical Science, Central South University, Changsha, China; ^8^ IMD Berlin, Institute of Medical Diagnostics, Berlin, Germany

**Keywords:** maternal and paternal programming, eNOS, metabolomics, sex-dependent effects, offspring

## Abstract

**Background:** Preclinical animal studies and clinical studies indicate that both maternal as well as paternal genetic alterations/gene defects might affect the phenotype of the next-generation without transmissions of the affected gene. Currently, the question of whether the same genetic defect present in the mother or father leads to a similar phenotype in the offspring remains insufficiently elucidated.

**Methods:** In this head-to-head study, we crossbred female and male mice with heterozygous endothelial eNOS knockout (eNOS+/−) with male and female wild-type (wt) mice, respectively. Subsequently, we compared the phenotype of the resulting wt offspring with that of wt offspring born to parents with no eNOS deficiency.

**Results:** Wt female offspring of mothers with heterozygous eNOS showed elevated liver fat accumulation, while wt male offspring of fathers with heterozygous eNOS exhibited increased fasting insulin, heightened insulin levels after a glucose load, and elevated liver glycogen content. By quantitative mass-spectrometry it was shown that concentrations of six serum metabolites (lysoPhosphatidylcholine acyl C20:3, phosphatidylcholine diacyl C36:2, phosphatidylcholine diacyl C38:1, phosphatidylcholine acyl-alkyl C34:1, phosphatidylcholine acyl-alkyl C36:3, and phosphatidylcholine acyl-alkyl C42:5 (PC ae C42:5) as well as four liver carbon metabolites (fructose 6-phosphate, fructose 1,6-bisphosphate, glucose 6-phosphate and fumarate) were different between wt offspring with eNOS+/− mothers and wt offspring with eNOS+/− fathers. Importantly, fumarate was inversely correlated with the liver fat accumulation in female offspring with eNOS+/− mothers and increased liver glycogen in offspring of both sexes with eNOS+/− fathers. The qRT-PCR results revealed that the gene expression patterns were different between wt offspring with eNOS+/− mothers and those offspring with eNOS+/− fathers. Different gene expression patterns were correlated with different observed phenotypic changes in male/female offspring born to mothers or fathers with a heterozygous eNOS genotype.

**Conclusion:** The identical parental genetic alteration (heterozygous eNOS deficiency), without being passed on to the offspring, results in distinct metabolic, liver phenotype, and gene expression pattern variations depending on whether the genetic alteration originated from the father or the mother.

## Introduction

The “fetal programming” hypothesis suggests that during a crucial early stage of life, specific environmental and nutritional factors can lead to permanent changes in organ structure and function in response to environmental influences. These alterations may, in turn, increase the risk of developing cardiovascular and metabolic diseases later in life (Reichetzeder et al., 2016). The fundamental factors contributing to fetal programming encompass maternal undernourishment during pregnancy, overnutrition, obesity, diabetes, and exposure to harmful toxins (Reichetzeder et al., 2016). A novel mechanism, initially proposed by Hocher et al. (2000) and subsequently validated by other researchers (Masuda et al., 2002; van Beynum et al., 2006; Tsai et al., 2008; Miodovnik et al., 2012; Warrington et al., 2019), suggests that maternal genes may influence the fetal phenotype independently of the fetal genome. Similarly, existing studies have already suggested that paternal genes, even without being transmitted to the offspring, may also influence the phenotype of the offspring (Nelson et al., 2010; Chen et al., 2012; Li et al., 2016; Zhang et al., 2019; Liu et al., 2021; Zhang et al., 2022).

In our prior research, we conducted crossbreeding experiments involving female and male heterozygous eNOS knockout mice and male and female wt mice. We then compared the phenotype of the resulting wt offspring from these crosses with that of wt offspring born to parents who were both wt mice (Hocher et al., 2016; Hocher et al., 2022). We specifically selected eNOS knockout mice for these experiments due to the critical role that eNOS plays in regulating placental and vascular functions (Kulandavelu et al., 2012; Kusinski et al., 2012). Heterozygous eNOS deficiency has been conclusively linked to the development of an adverse intrauterine environment. Remarkably, this environmental factor can exert a significant influence on the vascular phenotype of offspring (Costantine et al., 2008). In addition, the presence of heterozygous eNOS deficiency in male mice can potentially lead to an unfavorable testicular microenvironment, primarily attributed to impaired testicular vascular function (Hocher et al., 2022). The findings from our prior research indicated that female offspring with wt genetics, born to mothers with heterozygous eNOS deficiency, developed fatty liver disease. Wt male offspring born to fathers with heterozygous eNOS deficiency exhibited elevated fasting insulin levels, as well as increased insulin levels following glucose ingestion (Hocher et al., 2016; Hocher et al., 2022). However, until now, we have not conducted a comprehensive study comparing the effects of maternal and paternal eNOS deficiency on genetically healthy offspring head-to-head. Our overarching goal is to gain a deeper understanding of phenotypic variations and uncover potential molecular mechanisms, particularly in relation to differences in glycemic control.

Metabolomics is a rapid, robust, and efficient research tool that analyses many small molecules of biochemical pathways in tissue, blood, urine, and other biological fluids (Hocher and Adamski, 2017; Lu et al., 2018; Yong-Ping et al., 2020). Metabolites are influenced by both endogenous regulatory mechanisms and the environment (Bachlechner et al., 2016; Feldman et al., 2017; Boone et al., 2019). In this study, we thus used metabolomics to gain a more profound understanding of the distinctions between genetically healthy offspring originating from either mother or father with heterozygous eNOS deficiency.

## Materials and methods

### Breeding and study protocol

The whole study protocol received approval from the animal welfare committee in Berlin, Germany, and was conducted in accordance with the relevant local institutional guidelines. ENOS knockout mice (strain B6.129P2-Nos3tm1Unc/J) and C57BL/6J control mice were sourced from Jackson Laboratories (Bar Harbour, ME). Animals were housed at a controlled environment (21°C ± 2°C, 50% ± 10% relative humidity and a 12:12h light-dark cycle) and had access to food and water *ad libitum*. A comprehensive description of the breeding procedure was shown in Supplementary Figure S1. Female wild-type (wt) mice were bred with male eNOS knockout mice (eNOS−/−) to produce F1 offspring with heterozygous eNOS (eNOS+/−) genotypes. Subsequently, female F1 mice with heterozygous eNOS knockout and male F1 mice with heterozygous eNOS knockout were once again bred with male and female wt mice, respectively. Heterozygous animals used for breeding of the F2 generation were all derived from different dams, i.e., siblings were not used. In addition, the F1 mice chosen for breeding the subsequent generation were matched in terms of age. Following parturition by the female, we promptly standardized the litter size to ten pups (comprising five males and five females). These newborns were nurtured until weaning at 21 days of age. Only the F2 wt offspring resulting from this breeding procedure were included in the study, and they were compared to wt offspring born to parents who were both wild type.

The male and female F2 offspring were raised for 24 weeks and subsequently underwent separate analyses, including measurements of birth weight, final body weight, and liver weight. Blood pressure was measured in the 24th week by using the tail-cuff method, as previously described (Quaschning et al., 2007). In the 21st week, an intraperitoneal glucose tolerance test (IPGTT) was conducted. In brief, the animals underwent an overnight fasting period, followed by intraperitoneal injection of 2 mg glucose per gram of body weight. Blood samples were then collected from the tail vein at 0, 15, and 60-min intervals to measure plasma glucose and insulin levels, following previously established protocols (Kim et al., 2010; Wang et al., 2010; Du et al., 2012). The trapezoid rule was used to determine the area under curve (AUC) for glucose and insulin concentrations in each animal.

### Liver morphology

Liver morphology was analyzed under the microscope using two different stainings: Hematoxylin and Eosin (H&E), and Red Oil Staining.

a) H&E Staining: for preparation, the livers were washed with phosphate-buffered saline (PBS) buffer, then fixed with 4% (w/v) paraformaldehyde and embedded in paraffin. 3 μm thick slices were obtained using a Microm HM230 Microtomy and then stained with H&E. We identified hepatic venules and their adjacent portal fields by the sinusoidal connection between them. Ten lobules of each slide were identified using a Zeiss Axiovert 100 microscope (200 x), photographed with a Leica EC3 digital camera, and saved using LAS EZ software (Leica Microsystems). We measured the linear lobular dimensions by determining the distance from the center of the hepatic vein to the center of three associated portal vein branches using ImageJ (version 1.410, NIH shareware). Subsequently, we calculated the mean radius of lobules for each animal.
b) Oil Red O Staining was done as described before (Koopman et al., 2001). 30 pictures were taken per sample using an Olympus (Shinjuku, JP) BH-2 microscope (200x) and a digital camera CFW-1310C (Scion Corporation, Frederick, MD). The lipid content and the density of lipid droplets were quantified with ImageJ (version 1.410, NIH shareware).

### Liver glycogen content

Liver tissue was incubated with 1N KOH (95°C, 30 min). Glycogen was precipitated using saturated sodium sulfate solution (Na_2_SO_4_) and 95% (v/v) ethanol and washed twice in 60% (v/v) ethanol. Resuspended glycogen was degraded with 0.1% (w/v) amyloglucosidase (Sigma-Aldrich, St. Louis, MO) in acetate buffer (0.2 M sodium acetate, 0.46% (v/v) acetic acid, pH 4.8) for 2 h at 40°C. Glucose concentration was measured photometrically using the Glucose (HK) Assay Kit (Sigma-Aldrich). Glycogen content was expressed in relation to liver weight.

### Pancreas morphology

a) H&E Staining: pictures of whole tissue slides and of every islet of Langerhans were taken using the Zeiss Axiovert 100 microscope (25x/200x) and the Leica EC3 digital camera. The islets were counted, and the islet area was measured using ImageJ software to calculate the islet density and the mean islet area per slide.
b) Pancreas immunohistology: The beta cell content of islets of Langerhans was measured using immunohistological staining of insulin. We used an antibody against insulin (1:200, ab181547, abcam, Cambridge, United Kingdom) and a secondary antibody (1:500, ab97051, abcam, Cambridge, United Kingdom) diluted in antibody diluent (Dako, Glostrup, DK), and for visualization the ABC staining system (sc 2023, Santa Cruz Biotechnology, Santa Cruz, CA) following the instructions provided by the manufacturer. All islets per slide were photographed using an Olympus BH-2 microscope (200x) and a CFW-1310C digital camera. Thirty images for each sample were taken. The islet area and beta cell content were measured using ImageJ. The average islet size was obtained by total islet area/islet amount in each sample. The beta cell content was determined by the insulin positive staining area.

### Metabolomic profiles in serum

At the end of the experiment, blood was obtained via retro-orbital collection. The blood samples were then centrifuged at 3,000 × g for 10 min at 4°C to obtain serum. Afterwards, we analysed serum metabolomic profiles using the Absolute IDQTM p150 Kit (BIOCRATES Life Sciences AG, Innsbruck, Austria) and flow injection analysis-tandem mass spectrometry (FIA-MS/MS), following the kit’s instructions. Detailed information on the procedure of metabolite quantification has been previously provided (Lu et al., 2018; Yong-Ping et al., 2020). We quantified a total of 163 targeted metabolites simultaneously from 10 µL of serum. These included 92 glycerophospholipids [comprising 15 lysophosphatidylcholines (LPC) and 77 phosphatidylcholines (PC)], 40 acylcarnitines (acylC), free carnitine, 14 amino acids (13 of which were proteinogenic, plus ornithine), hexoses, and 15 sphingolipids (SM) (Yong-Ping et al., 2020). The quantification of metabolite concentrations [µM] was based on internal standards.

### Determination of central carbon metabolites in liver tissue

Liver tissues (15 to 30 mg each) were homogenized using a Fast Prep FP 120 homogenizer (Thermo Savant, Holbrook, NY) with 1 mL of phosphate buffer (pH 7.4) at a speed setting of 6.0, employing lysing matrix D. After homogenization, aliquots of the resulting homogenate were promptly frozen at −80°C until further analysis.

The concentrations of metabolites in the liver tissue homogenate were assessed using both gas chromatography-mass spectrometry (GC-MS) and liquid chromatography-tandem mass spectrometry (LC-MS-MS), following previously established protocols (Hofmann et al., 2008; Maier et al., 2010). We spiked defined volumes of liver homogenate (as outlined below) with an internal standard solution. These samples were then subjected to evaporation until dryness using a stream of nitrogen, followed by derivatization. Subsequently, we conducted the analysis using gas chromatography-mass spectrometry (GC-MS). The GC-MS analysis was carried out using a 5975C inert XL MSD, coupled with a 7890A GC from Agilent Technologies in the Electron Impact (EI) mode.

Concentrations of 3-hydroxybutyrate, malate, citrate, and fumarate were determined in 5 µL liver tissue homogenate using the respectively labeled analogs 3-hydroxy-[^2^H_4_] butyrate, [^13^C_4_] fumarate, [^13^C_4_] malate, and [^2^H_4_] citrate as internal standards. Following the evaporation step, the samples underwent derivatization to form methyloxime tert-butyldimethylsilyl derivatives (Hofmann et al., 2008).

To determine glucose 6-phosphate (G-6-P) and fructose 6-phosphate (F-6-P) concentrations, 25 µL liver homogenate was spiked with the internal standards ^13^C_6_-G-6-P and ^13^C_6_-F-6-P, evaporated to dryness, and derivatized to the trimethylsilyl derivatives.

Concentrations of ribose 5-phosphate (Rib-5-P), ribulose 5-phosphate/xylulose 5-phosphate (Ribu-5-P), sedoheptulose 7-phosphate (Sed-7-P), phosphoenolpyruvate (PEP), 6-phosphogluconate (6-PG), 2-/3-phosphoglycerate (3-PG), and fructose 1,6-bisphosphate (FBP) were determined in 10 µL of liver homogenate and analysed by LC-MS-MS as described (Maier et al., 2010).

### Quantitative real time PCR

We analysed a list of candidate genes involved in NO-synthase, metabolic process, energy homeostasis and fat storage, insulin-like growth factors and their binding proteins. RNA extraction from liver tissue and reverse transcription PCR were done like previously described (Hocher et al., 2022). Primers were obtained from Sigma-Aldrich, Eurofins (Ebersberg, GER) and shown in the Supplementary Table S1. The PCR was carried out on a Mx3000P thermal cycler (Stratagene, La Jolla, CA) with Power SYBR Green PCR Master Mix (Applied Biosystems, Foster City, CA), Sensi Mix or SensiFast low ROX kit (Bioline, London, United Kingdom) in accordance with the instructions. All samples were analysed in triplicate. The relative quantity of analysed genes was determined with the ΔΔCt method as described before (Hocher et al., 2022).

### Statistics

Statistical analysis was performed using SPSS version 22.0. All values are presented as mean ± SEM. For all datasets, we applied a two-way analysis of variance (two-way ANOVA), followed by *post hoc* Tukey test. Metabolomics data of serum were analysed using MetaboAnalyst 3.0. To mitigate the false discovery rate (FDR), we employed the Benjamini–Hochberg (BH) procedure to adjust *p*-values. The BH procedure is defined as follows: Pm ≤ (m/M) × q, where “m” represents the rank of a given *p*-value, “M” is the total number of tests (M = 163), and “q” is the desired FDR threshold (set up at 0.05 in this study) (Madar and FastLSU, 2016). For normally distributed data, we used the Pearson correlation analysis. Statistically significant differences were defined as *p*-value ≤ 0.05.

## Results

### Parental eNOS deficiency had no impact on birth weight, body weight, organ weight, and blood pressure of wt offspring

Male and female wt offspring with either eNOS+/− fathers or eNOS+/− mothers showed no significant differences in birth weight, final body weight, relative liver weight, or blood pressure when compared to the control group (Supplementary Table S2).

### Paternal eNOS deficiency led to an increase in fasting plasma insulin in male wt offspring

No differences in fasting plasma glucose were observed in male or female offspring born to eNOS+/− mothers/fathers. A significant increase in fasting plasma insulin was observed only in male wt offspring with eNOS+/− fathers (Supplementary Table S2).

### Paternal eNOS deficiency led to increased plasma insulin after glucose intake in male wt offspring

**FIGURE 1:**
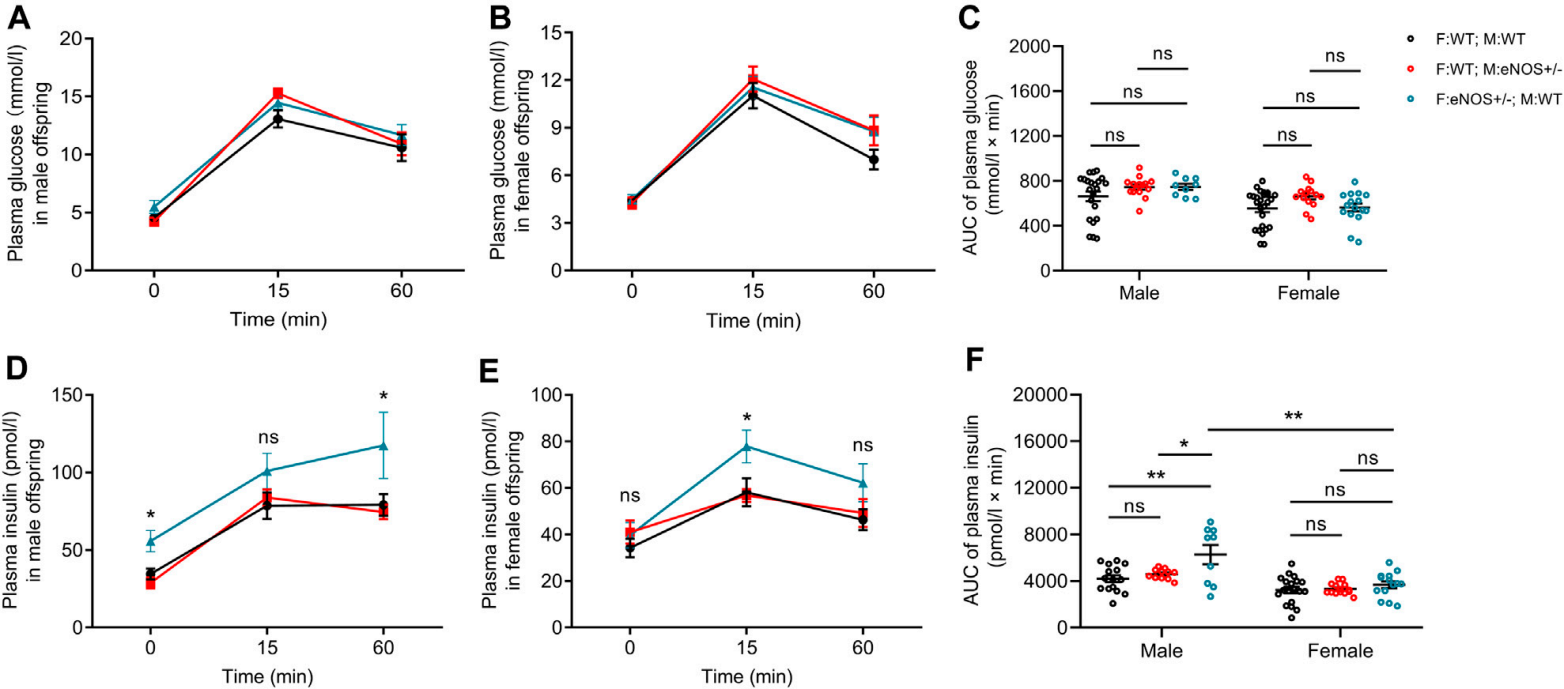
Comparison of plasma glucose **(A,B)** and insulin concentrations **(D,E)** during IPGTT in male **(A,D)** and female **(B,E)** offspring; AUC for IPGTT plasma glucose **(C)** and IPGTT plasma insulin **(F)** in male and female offspring. Black: F:WT; M:WT: wildtype offspring of wildtype fathers and wildtype mothers (male: *n* = 22 and female: *n* = 28); Red: F:WT; M: eNOS+/−: wildtype offspring of wildtype fathers and eNOS heterozygous mothers (male: *n* = 15 and female: *n* = 14); Blue: F: eNOS+/−; M:WT: wildtype offspring of eNOS heterozygous fathers and wildtype mothers (male: *n* = 9 and female: *n* = 14). The data were presented as mean ± SEM and analysed by two-way ANOVA followed by *post hoc* Tukey test. *p < 0.05; **p < 0.01; ns: *p* > 0.05.

IPGTT result showed that no differences in glucose concentrations at different time points after glucose intake and area under curve (AUC) of glucose in male or female offspring with eNOS+/− fathers/mothers (Figures 1A–C). Regarding insulin concentration, elevated insulin levels were observed at the 0 and 60 min after glucose intake in male offspring with eNOS+/− fathers (Figure 1D). Higher insulin levels at the 15 min after glucose intake were observed in female offspring with eNOS+/− fathers (Figure 1E). In addition, male offspring with eNOS+/− fathers had a significantly higher AUC of insulin (Figure 1F). No differences in insulin concentrations at different time points after glucose intake and AUC of insulin could be observed in male or female offspring born to eNOS+/− mothers.

### Parental eNOS deficiency affected liver morphology of wt offspring

**FIGURE 2:**
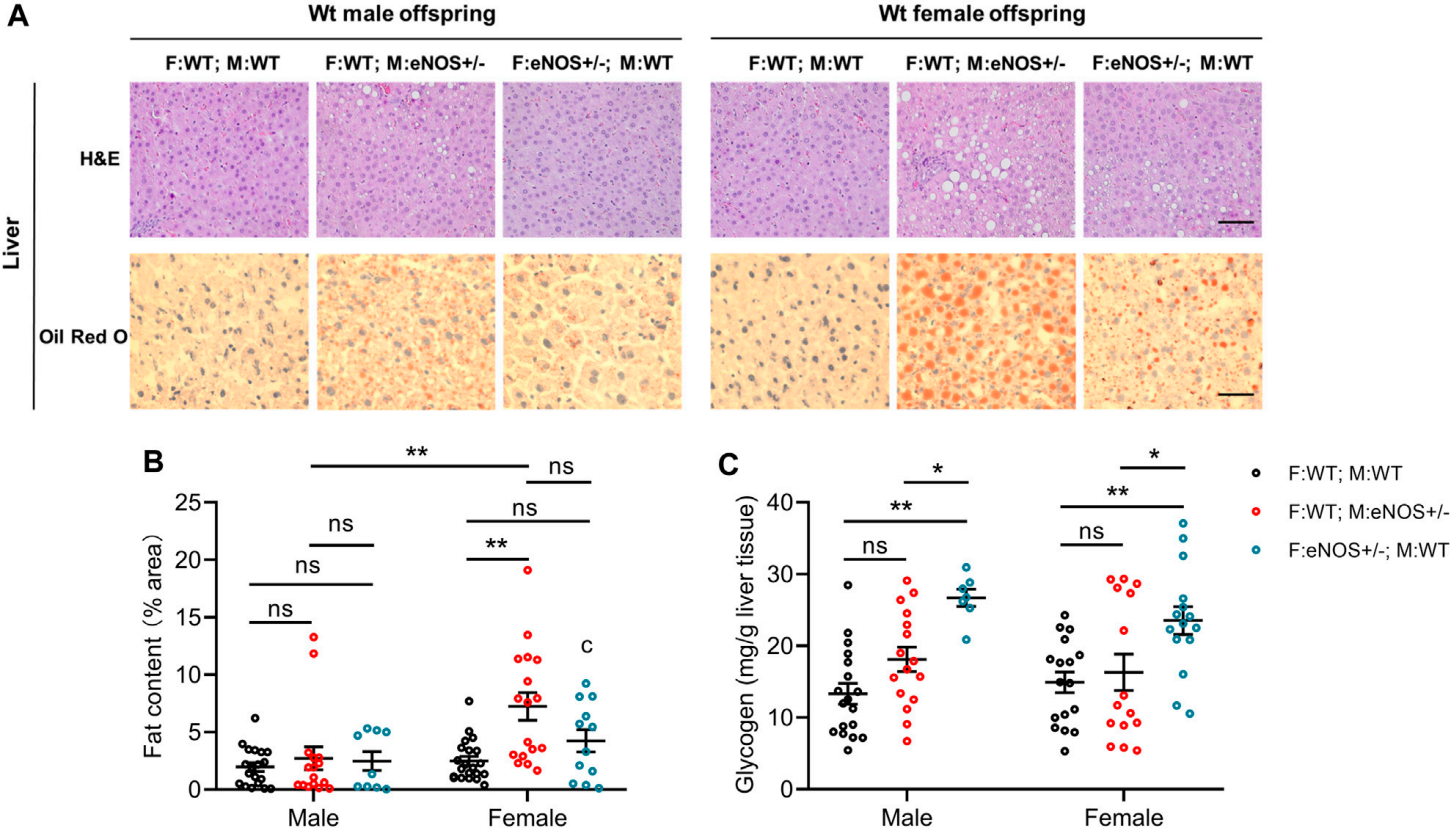
Morphological analysis of liver tissues. **(A)**: Representative images of haematoxylin and eosin (H&E) and Oil red O staining of liver sections (magnification: ×200 and scale bar: 50 μm); **(B)**: fat content and **(C)**: glycogen in the liver of wt offspring. Black: F:WT; M:WT: wildtype offspring of wildtype fathers and wildtype mothers (male: *n* = 19 and female: *n* = 21); Red: F:WT; M: eNOS+/−: wildtype offspring of wildtype fathers and eNOS heterozygous mothers (male: *n* = 16 and female: *n* = 17); Blue: F: eNOS+/−; M:WT: wildtype offspring of eNOS heterozygous fathers and wildtype mothers (male: *n* = 9 and female: *n* = 12). The data in **(B)** and **(C)** were presented as mean ± SEM and analysed by two-way ANOVA followed by *post hoc* Tukey test. *p < 0.05; **p < 0.01; ns: *p* > 0.05.

Liver lobule dimensions were consistent and similar among all groups. A significantly higher lipid droplet density and liver fat content were observed in female offspring born to eNOS+/− mothers (Supplementary Table S2; Figures 2A, B). However, the liver glycogen content was significantly higher in animals of both sexes born to eNOS+/− fathers (Figure 2C).

### Parental eNOS deficiency had no impact on pancreas morphology of wt offspring

The size and density of pancreatic islets of Langerhans, as well as the beta-cell content within the islets, exhibited no significant differences among all groups (Supplementary Figure S2; Supplementary Table S2).

### Parental eNOS deficiency led to differing metabolomic profiles in the serum of wt offspring

**FIGURE 3:**
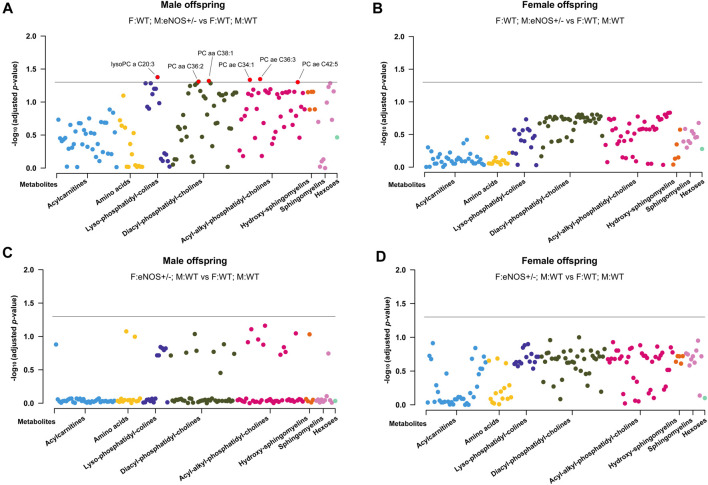
Manhattan Plot of offspring serum metabolites. **(A)** and **(B)**: serum metabolites in male **(A)** and female **(B)** wt offspring born to eNOS+/− mothers and wt fathers compared to those with wt parents; **(C)** and **(D)**: serum metabolites in male **(C)** and female **(D)** wt offspring born to eNOS+/− fathers and wt mothers compared to those with wt parents. The threshold line indicates the adjusted *p*-value of 0.05. F:WT; M:WT: wildtype offspring of wildtype fathers and wildtype mothers (male: *n* = 25 and female: *n* = 28); F:WT; M: eNOS+/−: wildtype offspring of wildtype fathers and eNOS heterozygous mothers (male: *n* = 13 and female: *n* = 18); F: eNOS+/−; M:WT: wildtype offspring of eNOS heterozygous fathers and wildtype mothers (male: *n* = 13 and female: *n* = 18). The *p*-values were adjusted by Benjamini–Hochberg (BH) procedure.

After adjusting the *p*-values using the Benjamini–Hochberg procedure, six metabolites (lysoPC a C20:3, PC aa C36:2, PC aa C38:1, PC ae C34:1, PC ae C36:3, and PC ae C42:5) of the 163 targeted serum metabolites were significantly reduced in male offspring born to eNOS+/− mothers and wt fathers (*p* < 0.05 and FDR < 0.05) (Figure 3). No metabolite was significantly different in female offspring with eNOS+/− mothers or eNOS+/− fathers and male offspring with eNOS+/− fathers (for more details see Supplementary Table S3).

### Parental eNOS deficiency led to differing liver carbon metabolites in liver tissue of wt offspring

**FIGURE 4:**
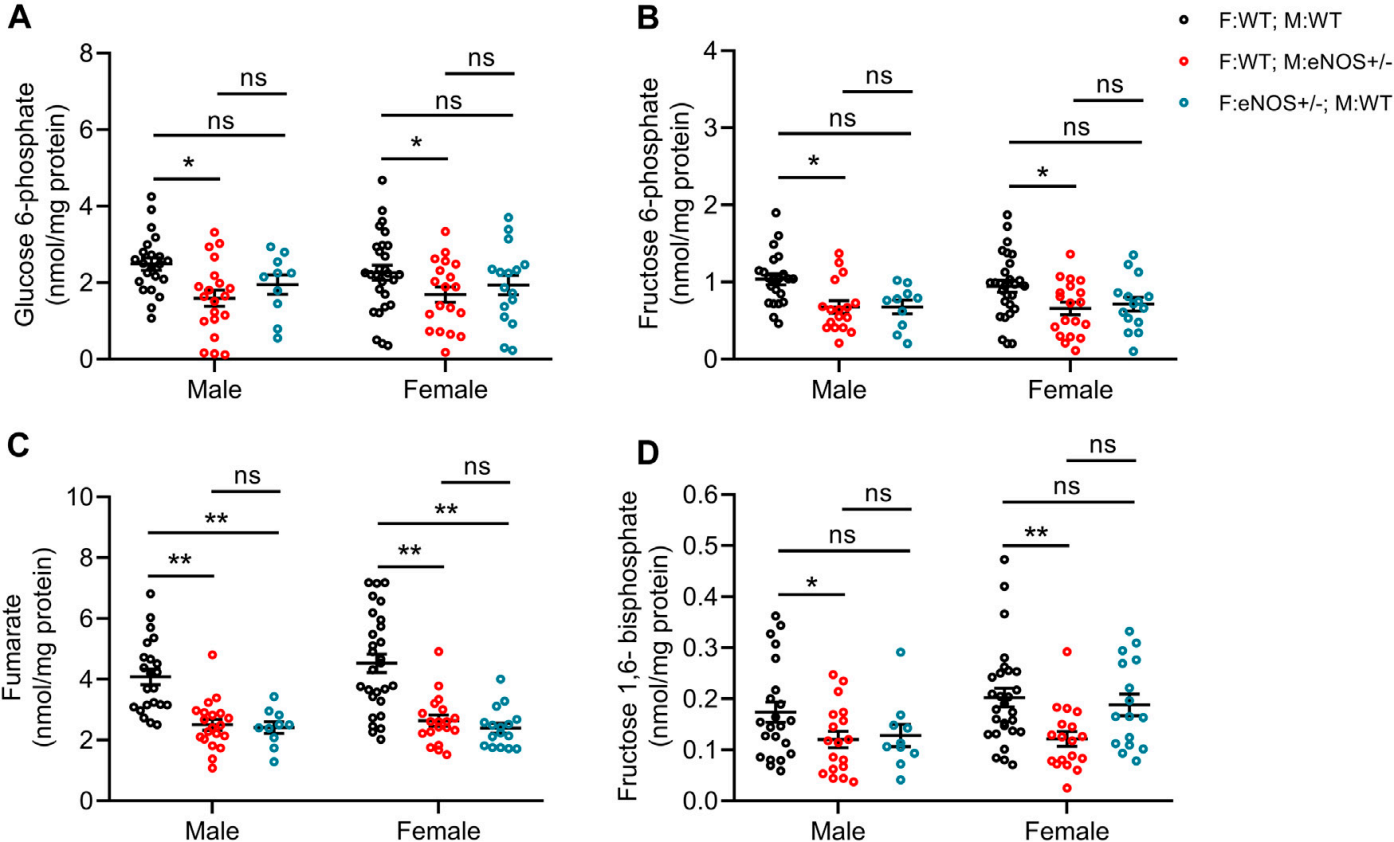
Comparison of carbon metabolite concentrations in liver tissue of offspring. **(A)**: glucose 6-phosphate; **(B)**: fructose 6-phosphate; **(C)**: fumarate; **(D)**: fructose 1,6-bisphosphate. Black: F:WT; M:WT: wildtype offspring of wildtype fathers and wildtype mothers (male: *n* = 21 and female: *n* = 27); Red: F:WT; M: eNOS+/−: wildtype offspring of wildtype fathers and eNOS heterozygous mothers (male: *n* = 16 and female: *n* = 17); Blue: F: eNOS+/−; M:WT: wildtype offspring of eNOS heterozygous fathers and wildtype mothers (male: *n* = 9 and female: *n* = 15). The data were presented as mean ± SEM and analysed by two-way ANOVA followed by *post hoc* Tukey test. *p < 0.05; **p < 0.01; ns: *p* > 0.05.

We quantified selected substrates of glucose metabolism in liver tissue using both GC-MS and LC-MS-MS technology. In both male and female offspring with eNOS+/− mothers, we observed significantly lower concentrations of fructose 6-phosphate, fructose 1,6-bisphosphate, glucose 6-phosphate and fumarate. Regarding the offspring with eNOS+/− fathers, both male and female offspring displayed significant reduction in fumarate concentration (Figure 4; Supplementary Table S4).

### Correlation analysis showed the relationship between altered metabolites in liver/serum and phenotype in wt offspring

**FIGURE 5:**
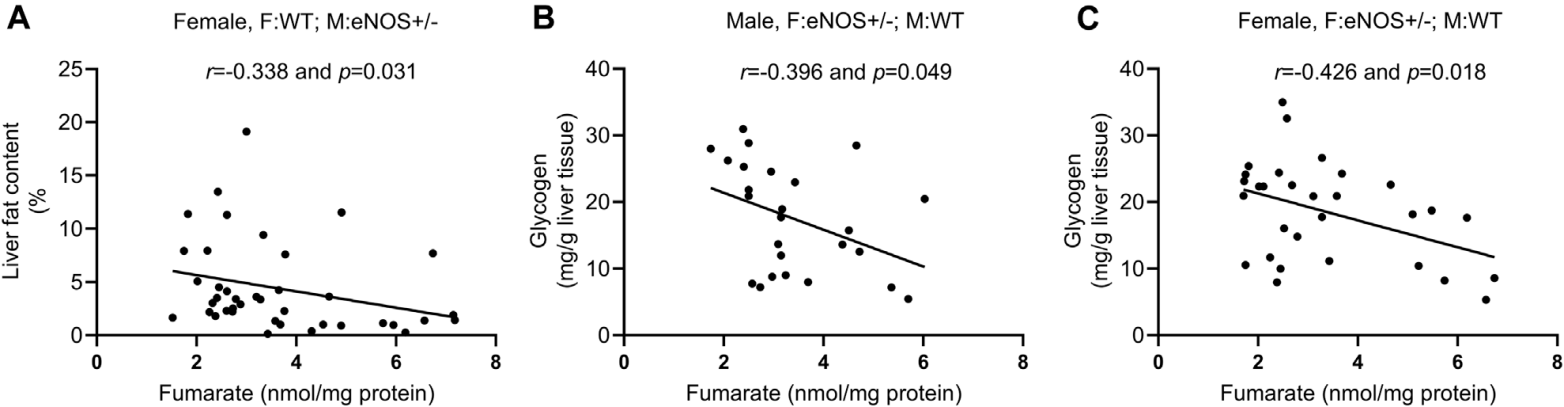
Correlation analysis between fumarate and phenotypic alterations in wild type offspring with eNOS+/− mothers/fathers. **(A)**: correlation between fumarate and liver fat content in female wt offspring with eNOS+/− mothers; **(B)** and **(C)**: correlation between fumarate and liver glycogen in male **(B)** and female **(C)** wt offspring with eNOS+/− fathers. F:WT; M:WT: wildtype offspring of wildtype fathers and wildtype mothers; F:WT; M: eNOS+/−: wildtype offspring of wildtype fathers and eNOS heterozygous mothers; F: eNOS+/−; M:WT: wildtype offspring of eNOS heterozygous fathers and wildtype mothers.

In wt offspring with eNOS+/− mothers, glucose 6-phosphate was negatively correlated with fat content in male offspring (*r* = −0.401, *p* < 0.05). Glucose 6-phosphate (*r* = 0.393, *p* < 0.05), fructose 6-phosphate (*r* = 0.388, *p* < 0.05), and fructose 1,6- bisphosphate (*r* = 0.404, *p* < 0.05) were positively correlated with glycogen content in female offspring. Fumarate was negatively correlated with the liver fat content in female offspring (*r* = −0.338, *p* < 0.05) (Figure 5A). LysoPC a C20:3 was positively correlated with the AUC of plasma insulin in female offspring (*r* = 0.448, *p* < 0.05). PC ae C42:5 was positively correlated with fat content in male offspring (*r* = 0.368, *p* < 0.05) (for more details see Supplementary Table S5).

In wt offspring with eNOS+/− father, fumarate was negatively correlated with glycogen content in both sexes (*r* = −0.396, *p* < 0.05; *r* = −0.426, *p* < 0.05) (Figures 5B, C). LysoPC a C20:3 had a positive correlation with fat content in male offspring (*r* = 0.394, *p* < 0.05) and glycogen content in female offspring (*r* = 0.375, *p* < 0.05). PC aa C38:1 was negatively correlated with glycogen content in male offspring (*r* = −0.459, *p* < 0.05). LysoPC a C20:3 (*r* = 0.460, *p* < 0.01), PC aa C38:1 (*r* = 0.404, *p* < 0.05), and PC ae C34:1 (*r* = 0.409, *p* < 0.05) were positively correlated with AUC of plasma insulin in female offspring (for more details see Supplementary Table S6).

### Parental eNOS deficiency resulted in different gene expression patterns in liver tissue of wt offspring

The qRT-PCR results revealed that the gene expression patterns were different between wt offspring with eNOS+/− mothers and those offspring with eNOS+/− fathers. In wt offspring with eNOS+/− mothers, PPARγ was significantly decreased while Pepck, Igf1, Igf2, Igfbp1 and Igfbp2 were significantly increased in male offspring. The expression of Srebf1c and Gck were significantly reduced while Fitm1 was significantly increased in female offspring. In wt offspring with eNOS+/− fathers, 12 genes were differentially expressed in male offspring and 3 genes were differentially expressed in female offspring. See more details in the Supplementary Table S7.

### Correlation analysis showed the relationship between altered genes and phenotypic changes in wt offspring

**FIGURE 6:**
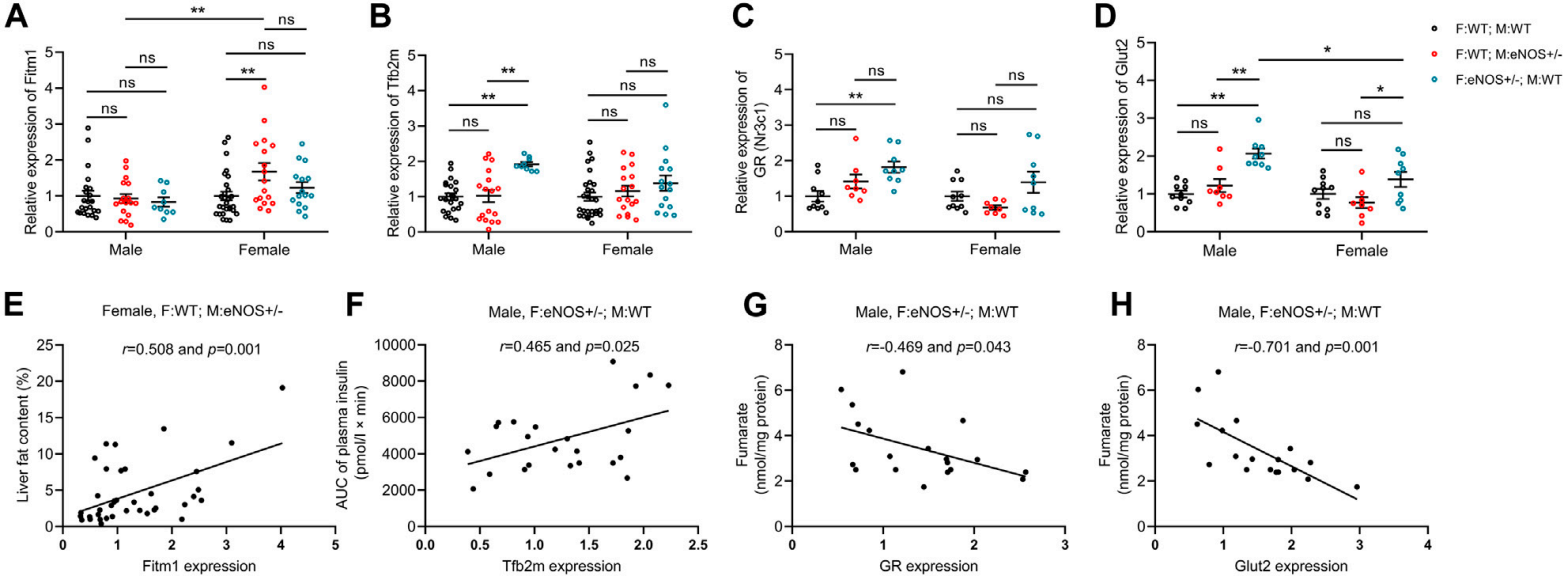
Gene expression (fold expression compared to control group) was analysed by real time PCR **(A–D)** and correlation analysis between altered genes and phenotypic changes **(E,F)** in wt offspring. **(G,H)**: correlation between altered genes and fumarate in male wt offspring with eNOS+/− fathers. Black: F:WT; M:WT: wildtype offspring of wildtype fathers and wildtype mothers (male: *n* = 22 and female: *n* = 28); Red: F:WT; M: eNOS+/−: wildtype offspring of wildtype fathers and eNOS heterozygous mothers (male: *n* = 17 and female: *n* = 17); Blue: F: eNOS+/−; M:WT: wildtype offspring of eNOS heterozygous fathers and wildtype mothers (male: *n* = 9 and female: *n* = 15). The data in **(A–D)** were presented as mean ± SEM and analysed by two-way ANOVA followed by *post hoc* Tukey test. *p < 0.05; **p < 0.01; ns: *p* > 0.05.

As shown in Figure 6, the liver fat ratio showed a significant correlation with the expression of Fitm1 (*r* = 0.508, *p* < 0.01) in female offspring with eNOS+/− mothers. AUC of plasma insulin positively correlated with Tfb2m expression (*r* = 0.465, *p* < 0.05) in male offspring with eNOS+/− fathers. Seven genes (Chrebp, GR, Tfam, Tfb2m, G6Pase, Glut2 and Igf2) showed a significant correlation with increased liver glycogen in male offspring with eNOS+/− fathers (Supplementary Table S9). Fumarate negatively correlated with the expression of GR (*r* = −0.469, *p* < 0.05) and Glut2 (*r* = −0.701, *p* < 0.01) in male offspring with eNOS+/− fathers. See more details in the Supplementary Tables S8, S9.

## Discussion

**FIGURE 7:**
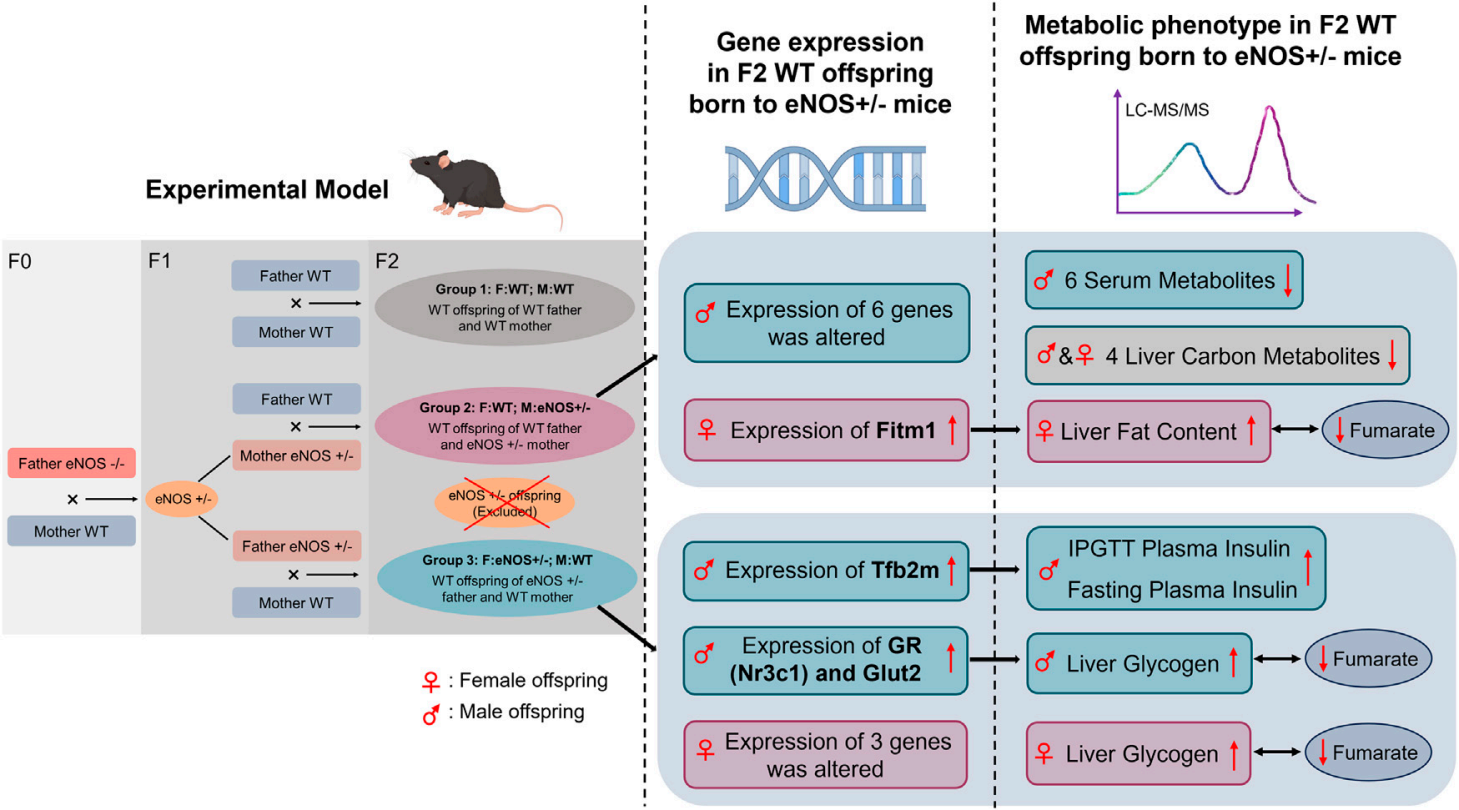
Graphical summary of the results obtained in this study. WT, Wild type; IPGTT, Intraperitoneal glucose tolerance test.

To investigate the advanced fetal programming hypothesis, which suggests that both maternal and paternal genes can influence the offspring’s phenotype without the direct transmission of parental genes to the offspring, we employed a similar approach to our previous study. We crossed female and male heterozygous eNOS knockout mice with male and female wild type mice. Then, we conducted a head-to-head study to simultaneously analyse the paternal and maternal effects on the offspring’s phenotype. Our study findings indicate that female wt offspring born to eNOS+/− mothers showed elevated liver fat accumulation. In contrast, male wt offspring born to eNOS+/− fathers displayed increased levels of fasting insulin, higher insulin levels following glucose intake, and elevated liver glycogen content (Figure 7). In addition, female wt offspring born to eNOS+/− fathers also showed an increased liver glycogen content. Pancreas morphology, including the endocrine pancreas, was not affected by parental eNOS deficiency. Our study identified six serum metabolites [lysoPhosphatidylcholine acyl C20:3 (lysoPC a C20:3), phosphatidylcholine diacyl C36:2 (PC aa C36:2), phosphatidylcholine diacyl C38:1 (PC aa C38:1), phosphatidylcholine acyl-alkyl C34:1 (PC ae C34:1), phosphatidylcholine acyl-alkyl C36:3 (PC ae C36:3), and phosphatidylcholine acyl-alkyl C42:5 (PC ae C42:5)] in male wt offspring and four liver carbon metabolites (fructose 6-phosphate, fructose 1,6-bisphosphate, glucose 6-phosphate and fumarate) in both sexes of wt offspring born to eNOS+/− mothers were significantly changed compared with those in wt offspring born to eNOS+/− fathers. These observations might be attributed to the adverse intrauterine environment in eNOS+/− mothers. Notably, further correlation analyses showed fumarate was inversely correlated with the fat accumulation in the liver in female offspring with eNOS+/− mothers and increased liver glycogen in offspring of both sexes with eNOS+/− fathers (Figure 7). Gene expression analysis revealed an elevated expression of liver Fitm1 (fat storage inducing transmembrane protein 1) in female offspring with maternal eNOS deficiency, which was associated with increased liver fat accumulation in these offspring. Importantly, our study revealed that the expression of the Tfb2m (mitochondrial transcription factor B2) gene was increased in male offspring born to fathers with eNOS deficiency, which was significantly correlated with elevated insulin levels after glucose load in these male offspring. Seven genes were significantly correlated with increased liver glycogen in in male offspring born to fathers with eNOS deficiency. Furthermore, in these genes, the increased expression of GR (glucocorticoid receptor) and Glut2 (glucose transporter 2) genes was significantly correlated with the decreased levels of fumarate in male offspring with paternal eNOS deficiency (Figure 7).

Our data align with recent research (Nelson et al., 2010; Liu et al., 2021; Chen et al., 2022; Zhao et al., 2022), which suggests that maternal or paternal genes, even without direct transmission to the offspring, can influence the offspring’s phenotype. This influence appears to depend on whether the gene defect was present in the mother or the father. One study investigated the transgenerational genetic effects of the fathers’ Y chromosome on daughters’ phenotypes and revealed that certain traits on the paternal Y chromosome (not inherited to daughters) significantly reduced anxiety levels among daughters (Nelson et al., 2010). Another study demonstrated that the mutation of paternal Usp26 (ubiquitin-specific peptidase 26) increased the risk of having children with Klinefelter syndrome (Liu et al., 2021). A recent study showed that the maternal environment affects offspring by influencing the level of oocyte TET3 (tet methylcytosine dioxygenase 3). This, in turn, has an impact on the reprogramming of the paternal genome within the zygote. The impairment of DNA demethylation and epigenetic inheritance specifically influences the expression of certain paternally hypermethylated genes involved in insulin secretion, including Gck (glucokinase), which plays a critical role in glucose metabolism. Consequently, this sensitizes the offspring to glucose intolerance (Chen et al., 2022). In addition, another study showed that deficiency of maternal Ezh1/2 (enhancer of zeste homolog 1/2) caused compromised H3K27me3 (tri-methylation of lysine 27 on histone H3 protein) and affected pluripotent epiblast cells within late blastocysts, which subsequently results in impaired embryonic development (Zhao et al., 2022). To sum up, genetic changes in the parental germline can affect the offspring’s phenotype, even if those changes are not encoded in the DNA sequence of the offspring. These changes can be influenced by many factors such as diet, stress, and exposure to toxins. The molecular mechanisms underlying these epigenetic effects induced by parental genes may involve DNA methylation, histone modification, or mediation through small RNAs.

The key molecule in this study is eNOS, an enzyme that produces nitric oxide (NO) in endothelial cells. NO plays a crucial role in locally regulating vascular resistance, promoting angiogenesis, and is also considered a potential regulator of placental steroid biosynthesis and nutrient uptake (Vatish et al., 2006). eNOS deficiency is linked to the occurrence of intrauterine growth retardation (IUGR) due to impaired placental blood flow and nutrient delivery (George et al., 2022). Parental stimuli, characterized by heterozygous eNOS deficiency, yield diverse phenotypic outcomes in their offspring, contingent upon the sex of the parent carrying the deficiency, while not being passed on to the subsequent generation. Female offspring of mothers with the eNOS+/− genotype exhibited heightened liver fat accumulation, while male offspring of fathers with the same genotype showed increased fasting insulin levels and enhanced liver glycogen storage. These distinct outcomes arising from the identical parental genetic defect (eNOS deficiency) may be attributed to varying effects of eNOS deficiency on the reproductive organs of males and females. Maternal eNOS deficiency can impact various aspects of reproductive and maternal physiology, including egg maturation, intrauterine development, nursing behavior, and lactation (Pallares et al., 2008; Teichert et al., 2008). It is important to note that maternal eNOS deficiency has negative effects on liver fat ratio most likely due to the influence on oocyte/intrauterine development induced by maternal eNOS deficiency. Paternal eNOS deficiency can have far-reaching effects, potentially influencing both the maturation and development of sperm (Mostafa et al., 2015). Furthermore, it may trigger transmissible epigenetic alterations within these sperm, giving rise to enduring epigenetic changes. These modifications can ultimately result in an adult phenotype characterized by elevated fasting insulin levels, heightened insulin response following glucose intake, and increased liver glycogen content. This explanation aligns with previous research that demonstrates how a paternal high-fat diet before conception can lead to impaired glucose tolerance in offspring, primarily due to epigenetic modifications in sperm and consequential alterations in target organs (Ng et al., 2010; Terashima et al., 2015; Chen et al., 2016; Nembhard et al., 2018), which correspond to our findings. In our prior research endeavours, we were able to establish disparities in both overall DNA methylation levels and gene-specific DNA methylation patterns, along with variations in the expression of specific candidate genes among wild-type offspring with either maternal or paternal eNOS deficiency. Notably, we observed a conspicuous correlation between DNA methylation patterns and the observed liver phenotype in these offspring (Hocher et al., 2016; Hocher et al., 2022). In the current study, we showed that hepatic fat accumulation in female wt offspring of eNOS+/− mothers was associated with increased expression of liver Fitm1. This gene was reported in our previous study (Hocher et al., 2016). However, we added the corresponding expression data of this gene in wt offspring born to eNOS+/− fathers in this study. In addition, we found that increased expression of the Tfb2m gene in male offspring born to fathers with eNOS deficiency was significantly correlated with elevated insulin levels after glucose load in these male offspring. Tfb2m is a mitochondrial transcription factor involved in mitochondrial DNA transcription. Existing evidence have showed that Tfb2m played a critical role in insulin secretion (Adan et al., 2008; Fex et al., 2018). Our observations were consistent with these findings. What’s more, we revealed that seven genes in male offspring born to fathers with eNOS deficiency were significantly correlated with increased liver glycogen in these male offspring. Particularly, we revealed the correlation among altered genes (GR and Glut2), metabolite (fumarate) and phenotypic changes (increased liver glycogen) in male offspring born to fathers with eNOS deficiency. These findings supplemented and added new evidence for a comprehensive understanding of how parental eNOS gene defects influence the phenotype of offspring, even when the offspring have not inherited the specific gene defect.

Sex differences on phenotypes were observed both in offspring of eNOS+/− mothers and of eNOS+/− fathers. Sexual dimorphism in fetal programming in response to identical stimuli has been extensively documented in previous studies (Reichetzeder et al., 2016). This divergence can be attributed to several factors. Firstly, both male and female sex steroid hormones, which are produced by both the fetus and the placenta, play a role in modulating the impact of mild NO deficiency on epigenetic and phenotypic changes in the offspring in a sex-specific manner. Additionally, sex-dependent transcriptional variations in the offspring may contribute to these observed differences (Bermejo-Alvarez et al., 2011; Li et al., 2016; Reichetzeder et al., 2016).

In the present study, we employed metabolomics to investigate further links between the observed offspring phenotypes and maternal/paternal eNOS deficiency. Characteristic metabolites associated with fatty liver disease and increased insulin and liver glycogen storage were identified. The precise pathophysiological significance of the serum metabolites we observed remains not fully elucidated. Phosphatidylcholines, including lyso-phosphatidylcholines, diacyl-phosphatidylcholines, and acyl-alkyl-phosphatidylcholines, are vital constituents of cell membranes and lipoproteins (Cole et al., 2012). Beyond their fundamental structural role, these molecules seem to play a role in various physiological processes. Notably, they are implicated in the liver’s secretion of very low-density lipoproteins and are also associated with glucose regulation (Cole et al., 2012; Furse and de Kroon, 2015; Boone et al., 2019). Phosphatidylcholines have also been demonstrated to enhance the cell proliferation effects of insulin and insulin-like growth factor-1 (Kiss, 1999). Furthermore, alterations in the concentrations of phosphatidylcholines were linked to cardiometabolic changes triggered by an excess of liver and visceral fat (Floegel et al., 2013) and atherosclerosis (Matsumoto et al., 2007). In line with these studies, we identified that lysoPC a C20:3 was specifically associated with the AUC of plasma insulin in female offspring born to eNOS+/− mothers/fathers. PC aa C38:1 and PC ae C34:1 had a positive relation with the AUC of plasma insulin in female offspring with eNOS+/− fathers. lysoPC a C20:3 was positively correlated with glycogen content in female offspring, while PC aa C38:1 was negatively correlated with glycogen content in male offspring born to eNOS+/− fathers. In summary, while the specific functions of the majority of metabolites observed in our study remain uncertain, there appears to be an association between these metabolites and alterations in plasma insulin levels, liver fat, and glycogen, induced by maternal or paternal eNOS deficiency.

Particularly, we found that fumarate had a significant correlation with increased fat accumulation in liver in female offspring born to eNOS+/− mothers and increased liver glycogen in offspring of both sexes born to eNOS+/− fathers. Fumarate plays a pivotal role as a key intermediate in the tricarboxylic acid cycle (TCA), facilitating the interconnection of carbon and nitrogen metabolism (Araujo et al., 2011; Hengist et al., 2019). One way in which fumarate can indirectly affect blood glucose levels is by influencing insulin signaling. Studies have shown that fumarate can enhance insulin sensitivity and improve glucose uptake by cells, which may help to lower blood glucose levels (Franko et al., 2022). Additionally, fumarate has been shown to activate AMP-activated protein kinase (AMPK) is a critical enzyme that plays a central role in the regulation of glucose metabolism (Li et al., 2011). With regards to fat accumulation, fumarate may indirectly impact this process by affecting mitochondrial function. Fumarate has been demonstrated to stimulate mitochondrial biogenesis and improve mitochondrial function, which may help to reduce fat accumulation (Wang et al., 2021). When it comes to glycogen, fumarate can impact glycogen storage in the liver indirectly through its effects on ATP production, AMPK activation, and insulin sensitivity (Noster et al., 2019). Consistent with these previous studies, our research also illustrated that fumarate was significantly related to the changes in liver fat accumulation and liver glycogen, induced by maternal/paternal eNOS+/− deficiency in offspring.

Our study has also several limitations. The primary goal of our study was to analyze potential sex-dependent effects of parental eNOS deficiency on the offspring’s phenotype and we indeed could demonstrate that it matters whether the parental heterozygous eNOS deficiency was present in the father or mother in our head-to-head study. However, sex dependent effects of the origin of homozygous eNOS deficiency in the grandfather/grandmother’s generation could also affect the phenotype in the F2 generation. There is some evidence that it would have affected the phenotype of the heterozygous eNOS mice coming from earlier studies by Longo M et al., showing that eNOS heterozygous offspring born to eNOS knockout mothers had higher blood pressure, effects on glucose tolerance and insulin levels compared to those offspring born to eNOS knockout fathers most likely because eNOS knockout mothers had an abnormal uterine environment (Longo et al., 2016). This uterine effect is absent when starting with homozygous eNOS deficient fathers. Therefore, we used male F0 eNOS−/− mice as origin of eNOS deficiency in our study. Given the study by Longo M et al., it is very likely that if the origin of eNOS deficiency would be the grandmother in the breeding protocol it would have affected the phenotype in the F2 generation, but this represents another research question that merits to be addressed in an independent study designed to address this topic. Epigenetic alterations of the adult phenotype are mainly caused by intrauterine epigenetic alterations of gene expression and subsequent alterations of the function and morphology of organs (Reichetzeder et al., 2016). This is clearly an important task for further studies to better understand the early life epigenetic mechanisms of parental eNOS deficiency. In addtion, we measured insulin and glucose levels at 0, 15, and 60 min during the IPGTT, with no further measurements to prioritize animal welfare. However, the duration of 60 min for IPGTT demonstrated differences among the groups and was also reported in previous studies (Kim et al., 2010; Wang et al., 2010; Hocher et al., 2022). What’s more, the specific metabolomics platform used in this study restricted our choice of metabolites to investigate. On the other hand, an advantage of this platform is that it includes a collection of metabolites that are both biologically and analytically well-defined. Another limitation of our study is the absence of an analysis on the potential impact of eNOS heterozygosity on the metabolism of the parents (F1 generation) and an analysis on the hererozygous eNOS fetuses of F2 genetation. Some other studies already provided evidence on phenotype of eNOS heterozygous mice. Cook et al. have shown that eNOS^−/+^ heterozygous mice were normotensive and had normal insulin sensitivity on a normal diet (Cook et al., 2004). Consequently, we did not investigate further on the metabolic phenotype of F1 eNOS heterozygous mice and mainly focused on the metabolic changes observed in the F2 generation with a healthy genotype. Additionally, we did not analyse the underlying epigenetic alterations. However, this was not done, because it was described recently by us using comparable experimental designs of the animal studies—increased liver glucocorticoid receptor and Ppargc1a gene expression attributed to altered methylation patterns of these genes when the father had eNOS deficiency and increased liver Fat Storage Inducing Transmembrane Protein 1 (*Fitm1*) and Cyclin-dependent kinase inhibitor 1A (Cdkn1a) gene expression resulted from altered methylation of these genes when the mother had eNOS deficiency (Kim et al., 2010; Wang et al., 2010; Hocher et al., 2022).

In conclusion, this head-to-head study demonstrated that the identical parental genetic modification (heterozygous eNOS deficiency) without transmission to the offspring causes an offspring metabolic and liver phenotype and liver gene expression pattern depending on whether the alteration was present in the father or the mother. Female offspring with wildtype genes from mothers with a heterozygous eNOS deficiency showed increased liver fat accumulation. In contrast, male offspring with wildtype genes from fathers with a heterozygous eNOS deficiency had higher fasting insulin levels, increased insulin response after a glucose load, and elevated liver glycogen content. We identified six serum metabolites and four liver carbon metabolites that differed between wt offspring with eNOS+/− mothers and wt offspring with eNOS+/− fathers. The most prominent effects were observed regarding fumarate (strong correlations between fumarate and changes on liver histology induced by maternal/paternal eNOS deficiency). Moreover, the gene expression patterns were different between wt offspring with eNOS+/− mothers and those offspring with eNOS+/− fathers. Importantly, the changes in specific gene expression were found to be correlated with the observed phenotypic alterations in wt offspring with eNOS+/− mothers/fathers. Our findings enhance the understanding of how parental genetic defects may impact the phenotype of genetically healthy offspring. This provides a foundation for more precise genetic counselling and screening. The clinical implications of our study should be further investigated in monogenic inherited diseases such as thalassemia.

## Data Availability

The original contributions presented in the study are included in the article/Supplementary Material, further inquiries can be directed to the corresponding author.
