# Supplementary material for: Parental sex-dependent effects of either maternal or paternal eNOS deficiency on the offspring’s phenotype without transmission of the parental eNOS deficiency to the offspring
Source: Front Physiol. 2023 Dec 19;14:1306178. doi: 10.3389/fphys.2023.1306178 (PMC10758467; doi:10.3389/fphys.2023.1306178)
Supplement: Supplementary file 1 [file DataSheet1.docx]

**Supplementary materials**


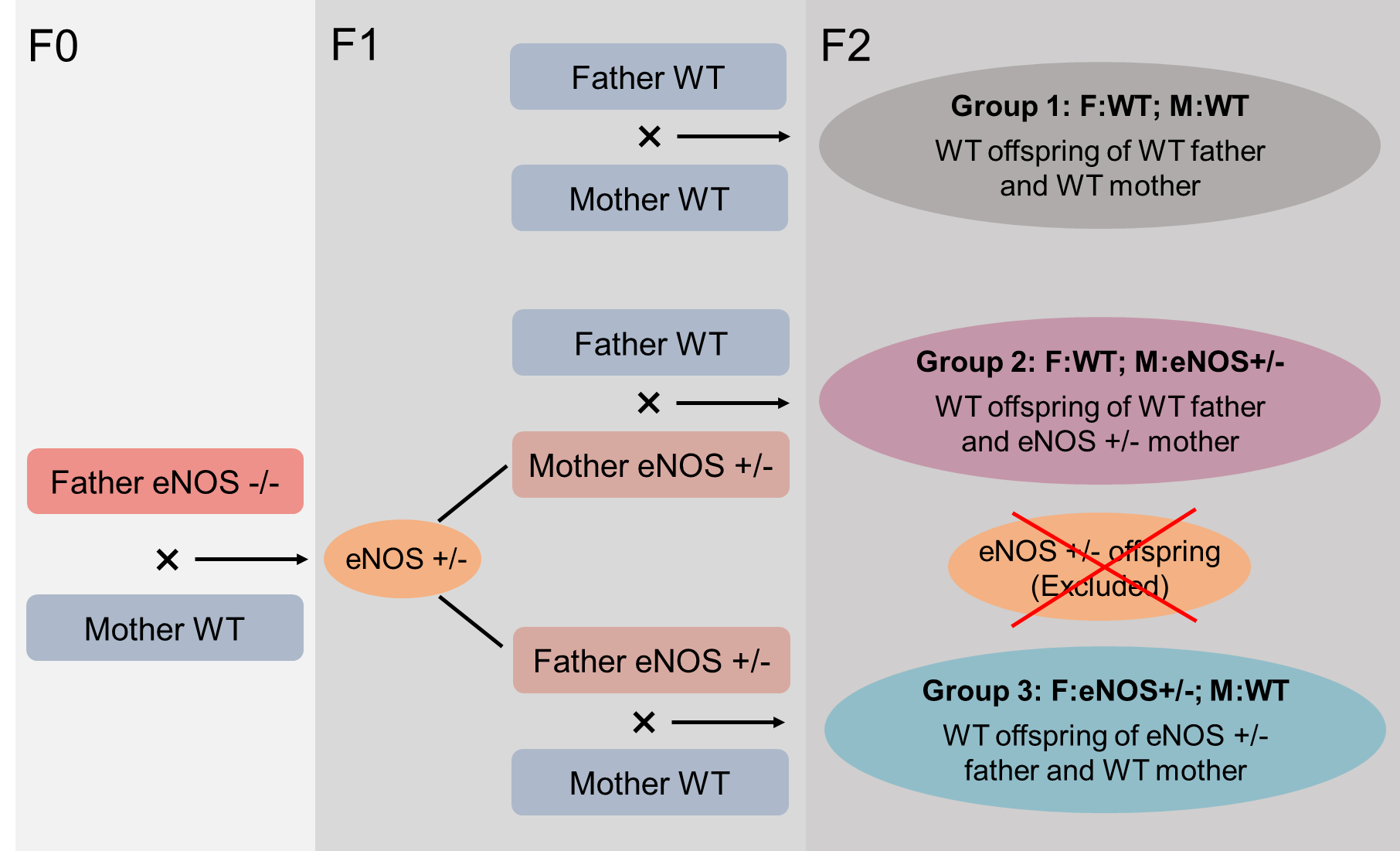


**Supplementary Figure 1. Study design for the F0, F1 and F2 generations**.

eNOS knockout mice of the C57BL/6J strain and their wild-type (wt) littermate were used. Female wt mice were cross-bred with homozygous male eNOS knockout mice. The resulting male and female heterozygous eNOS knockout (eNOS^+/-^) mice were then crossed with female and male wt mice, respectively, to produce the F2 generation. Only wt offspring of this breeding procedure (F2 generation) entered the study. These mice were compared to wt mice resulting from crossing male wt and female wt mice. Heterozygous animals used for breeding of the F2 generation were all derived from different dams i.e. siblings were not used.


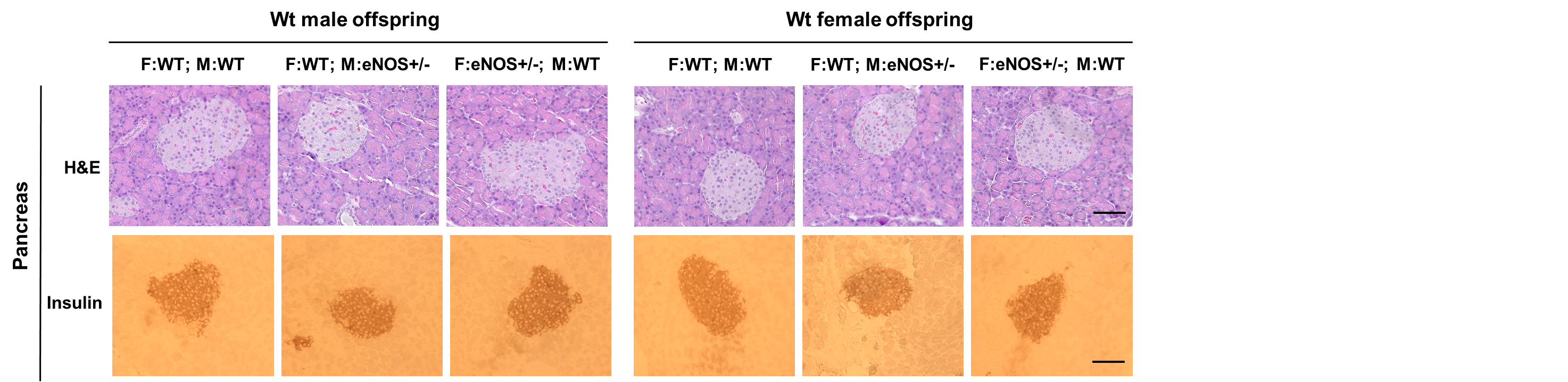


**Supplementary Figure 2. Morphological analysis of pancreas tissues.** Representative images of haematoxylin and eosin (H&E) and insulin staining of pancreas sections (magnification: 200× and scale bar: 50μm).

**Supplementary Table 1. Primers used for real time PCR**

| **Target Gene** | **Primer Pair** |
| --- | --- |
| eNOS (Nos3, ENSMUSG00000028978) | fw 5‘ GGGAAAGCTGCAGGTATTTG ’3  rev 5‘ GCTGAACGAAGATTGCCTCT '3 |
| iNOS (Nos2, ENSMUSG00000020826) | fw 5‘ TGACACACAGCGCTACAACA ’3  rev 5‘ CCATGATGGTCACATTCTGC ' 3 |
| Chrebp (Mlxipl, ENSMUSG00000005373) | fw 5‘ GAAGATGCTTATGTTGGCAATG ’3  rev 5‘ GGCGGTAATTGGTGAAGAAA ’3 |
| Srebf1c (ENSMUSG00000020538 ) | fw 5‘CTGTCGGGGTAGCGTCTG’3  rev 5‘CGGGAAGTCACTGTCTTGGT’3 |
| PPARα (Ppara, ENSMUSG00000022383) | fw 5’ TCTGGAAGCTTTGGTTTTGC '3  rev 5‘ TTCGACACTCGATGTTCAGG '3 |
| PPARγ (Pparg, ENSMUSG00000000440) | fw 5‘ CAGGCCTCATGAAGAACCTT’ 3  rev 5‘ GGATCCGGCAGTTAAGATCA’ 3 |
| PPARγ Co1α (Ppargc1a, ENSMUSG00000029167) | fw 5‘AGTCACCAAATGACCCCAAG’ 3  rev 5‘GGAGTTGTGGGAGGAGTTAGG’ 3 |
| GR (Nr3c1, ENSMUSG00000024431) | fw 5' ACACGTCAGCACCCCATAAT '3  rev 5' AGGCCGCTCAGTGTTTTCTA '3 |
| Tfam (ENSMUSG00000003923 ) | fw 5’ ACACCCAGATGCAAAACTTTC ‘3  rev 5’ CTTTGTATGCTTTCCACTCAGC ‘3 |
| Tfb1m (ENSMUSG00000036983) | fw 5’ AGGAAGTGGCTGAGAGACTTGT ‘3  rev 5’ GGTGCACCACTCCTACATCA ‘3 |
| Tfb2m (ENSMUSG00000026492) | fw 5’ CGATCTGTACTCCTGCGAATC ‘3  rev 5’ ACCAAGGTTCCATGTGCAG ‘3 |
| Nrf1 (ENSMUSG00000058440) | fw 5’ TCATCTCGTACCATCACAGACC ‘3  rev 5’ TTTGTTCCACCTCTCCATCAG ‘3 |
| Nampt (ENSMUSG00000020572) | fw 5’ CACCGACTCGTACAAGGTTACTC ‘3  rev 5’ TTTCACGGCATTCAAAGTAGG ‘3 |
| AMPK-α2 (Prkaa2, ENSMUSG00000028518) | fw 5‘AAAGACATACGAGAACATGAATGG’3  rev 5‘CTTCACAGCCTCATCGTCAA’3 |
| Fbp1 (FBPase)(ENSMUSG00000069805) | fw 5' ATCAAAGCCATCTCGTCTGC '3  rev 5' ATTTGCCCCTCTTCTCAGGT '3 |
| Gck (ENSMUSG00000041798) | fw 5' AAGTCCCACGATGTTGTTCC '3  rev 5' CTTCCCTGTAAGGCACGAAG '3 |
| Pepck (Pck1, ENSMUSG00000027513) | fw 5' ATACATGGTGCGGCCTTTC '3  rev 5' GACAACTGTTGGCTGGCTCT '3 |
| PK-L (Pklr, ENSMUSG00000041237) | fw 5’AGTATGGAAGGGCCAGCA ‘3  rev 5’AGAGGTGTTCCAGGAAGGTG ‘3 |
| G6Pase (G6pc, ENSMUSG00000078650) | fw 5' GACTGTGGGCATCAATCTCC '3  rev 5' TCACAGGTGACAGGGAACTG '3 |
| Glut 2 (Slc2a2, ENSMUSG00000027690) | fw 5’ GACGTCAATGGCACAGACAC ’3  rev 5’ GCCAACATTGCTTTGATCCT ’3 |
| FAS (Fasn, ENSMUSG00000025153) | fw 5‘ GGATTCGGTGTATCCTGCTG ’3  rev 5‘ TGGGCTTGTCCTGCTCTAAC ’3 |
| Acc1 (Acaca, ENSMUSG00000020532) | fw 5’ TTTCACATGAGATCCAGCATG ‘3  rev 5’ GCCACAGTGAAATCTCGTTG ‘3 |
| Cpt1a (ENSMUSG00000024900) | fw 5‘CGCACATTACAAGGACATGG ’3  rev 5‘TCTGCTCTGCCGTTGTTGT ’3 |
| HSL (Lipe, ENSMUSG00000003123) | fw 5‘ACCTGCTTGGTTCAACTGGA’3  rev 5‘CTGGCACCCTCACTCCATAG’3 |
| Fitm1 (ENSMUSG00000022215) | fw 5’ TGCTTACGGCGCCTCTAC ‘3  rev 5’CACAAACTTTATGTTGAAGAAGTTGC ‘3 |
| Fitm2 (ENSMUSG00000048486) | fw 5’ AAGCGCAACGTCCTCAAC ‘3  rev 5’ CAGATATACCAGATGGCTGTGC ‘3 |
| Igf1 (ENSMUSG00000020053 ) | fw 5' CGTCTTCACACCTCTTCTACCTG '3  rev 5' CCTGTGGGCTTGTTGAAGTAA '3 |
| Igf2 (ENSMUSG00000048583) | fw 5' TCTACTTCAGCAGGCCTTCAA '3  rev 5' GGGTATCTGGGGAAGTCGTC '3 |
| Igfbp1 (ENSMUSG00000020429) | fw 5' CAGCATGAAGAGGCAAAGG '3  rev 5' CTATAGGTGCTGATGGCGTTC '3 |
| Igfbp2 (ENSMUSG00000039323) | fw 5' AGGTCCTGGAGCGGATCT '3  rev 5' CATCTTGCACTGCTTAAGGTTG '3 |
| Igfbp3 (ENSMUSG00000020427) | fw 5' TGCTCCAGGAAACATCAGTG '3  rev 5' GGAGTGGATGGAACTTGGAA '3 |
| β-Actin (Actb, ENSMUSG00000029580) | fw 5' GATATCGCTGCGCTGGTC '3  rev 5' CATCACACCCTGGTGCCTA '3 |

**Supplementary Table 2. Birth weight, body weight, liver weight, systolic blood pressure, fasting plasma glucose, insulin, and histological findings in the liver and pancreas.**

| Variable | Male offspring | | | Female offspring | | |
| --- | --- | --- | --- | --- | --- | --- |
|  | F: WT; M: WT  (n=22) | F: WT; M: eNOS+/- (n=15) | F: eNOS +/-; M: WT (n=10) | F: WT; M: WT (n=24) | F: WT; M: eNOS+/- (n=18) | F: eNOS +/-; M: WT (n=14) |
| ***Birth weight, body weight, liver weight and systolic blood pressure*** | | | | | | |
| Birth weight (g) | 1.46±0.04 | 1.28±0.04 | 1.53±0.08 | 1.35±0.04 | 1.30±0.04 | 1.43±0.07 |
| Final body weight (g) | 33.91±0.89 | 31.15±0.82 | 31.67±1.83 | 21.82±0.44 | 23.31±0.64 | 23.41±0.65 |
| Liver weight (g) | 1.54±0.05 | 1.41±0.04 | 1.42±0.08 | 0.89±0.02 | 1.00±0.04 | 1.00±0.05 |
| Relative Liver Weight (% of body weight) | 4.56±0.08 | 4.56±0.10 | 4.49±0.09 | 4.09±0.08 | 4.29±0.07 | 4.19±0.14 |
| Systolic blood pressure (mmHg) | 95.63±2.90 | 95.16±3.31 | 99.81±1.55 | 109.29±3.14 | 105.06±2.75 | 109.94±2.50 |
| ***Fasting plasma glucose and insulin*** | | | | | | |
| Fasting glucose (mmol/L) | 4.52±0.35 | 4.60±0.32 | 5.43±0.59 | 3.98±0.20 | 4.60±0.30 | 4.86±0.38 |
| Fasting insulin (pmol/L) | 47.12±5.22 | 38.10±5.17 | 72.21±12.42^bc^ | 36.21±6.29 | 31.17±3.33 | 44.09±5.98 |
| **Histological findings in the liver** | | | | | | |
| Liver Lobular Dimension (mm) | 0.07±0.001 | 0.07±0.001 | 0.07±0.003 | 0.07±0.002 | 0.07±0.002 | 0.06±0.003 |
| Lipid droplet  Density (droplets/mm2) | 2239.75±747.54 | 2546.34 ±822.85 | 1585.9±569.46 | 3445.78±685.75 | 6624.64±1962.24^a^ | 3414.52±775.53^c^ |
| Fat Content (% area) | 1.96±0.38 | 2.71±1.00 | 2.47±0.83 | 2.49±0.38 | 7.24±1.20^a^ | 4.25±0.96 |
| Glycogen | 13.43±1.54 | 18.13±1.77 | 28.00±2.29^bc^ | 14.92±1.53 | 15.39±2.53 | 22.57±1.81^bc^ |
| ***Histological findings in the pancreas*** | | | | | | |
| Pancreatic island area (mm^2^) | 0.011±0.002 | 0.008±0.001 | 0.007±0.002 | 0.012±0.002 | 0.013±0.003 | 0.017±0.007 |
| Pancreatic island density (islands/cm^2^) | 116.50±14.21 | 143.58±13.41 | 128.08±14.41 | 126.48±13.65 | 100.02±11.00 | 134.85±16.65 |
| Pancreatic beta cell content (%) | 50.95±5.79 | 52.77±5.26 | 65.07±5.93 | 62.66±3.96 | 55.11±5.43 | 58.74±5.09 |

F:WT; M:WT: wildtype offspring of wildtype fathers and wildtype mothers; F:WT;M:eNOS+/-: wildtype offspring of wildtype fathers and eNOS heterozygous mothers; F:eNOS+/-;M:WT: wildtype offspring of eNOS heterozygous fathers and wildtype mothers. Data are given as mean ± SEM. a: p<0.05, F:WT; M:eNOS+/- vs F:WT; M:WT; b: p<0.05, F:eNOS+/-; M:WT vs F:WT; M:WT; c: p<0.05, F:eNOS+/-; M:WT vs F:WT; M:eNOS+/-.

**Supplementary Table 3. Metabolomic profiles in serum**

| Variable | Male offspring | | | Female offspring | | |
| --- | --- | --- | --- | --- | --- | --- |
| MetIQ Short Name | F: WT; M: WT  (n=25) (µM) | F: WT; M: eNOS+/-(n=13) (µM) | F: eNOS+/-; M: WT (n=13) (µM) | F: WT; M: WT (n=28) (µM) | F: WT; M: eNOS+/-(n=18) (µM) | F: eNOS+/-; M: WT (n=18) (µM) |
| **Acylcarnitines** | | | | | | |
| C0 | 31.18±11.2 | 24.3±11.69 | 25.72±8.54 | 29.45±11.38 | 29.37±12.04 | 28.83±11.89 |
| C10 | 0.1±0.03 | 0.09±0.02 | 0.1±0.03 | 0.09±0.02 | 0.09±0.03 | 0.1±0.03 |
| C10:1 | 0.08±0.02 | 0.09±0.02 | 0.09±0.02 | 0.08±0.02 | 0.08±0.02 | 0.09±0.02 |
| C10:2 | 0.03±0.01 | 0.03±0.01 | 0.03±0.01 | 0.03±0.01 | 0.03±0.01 | 0.03±0.01 |
| C12 | 0.06±0.03 | 0.07±0.05 | 0.06±0.04 | 0.05±0.04 | 0.06±0.04 | 0.05±0.02 |
| C12-DC | 0.05±0.01 | 0.05±0.01 | 0.05±0.01 | 0.06±0.01 | 0.06±0.01 | 0.05±0 |
| C12:1 | 0.1±0.03 | 0.11±0.04 | 0.12±0.04 | 0.11±0.04 | 0.11±0.03 | 0.12±0.02 |
| C14 | 0.11±0.09 | 0.17±0.14 | 0.12±0.11 | 0.09±0.09 | 0.11±0.09 | 0.08±0.06 |
| C14:1 | 0.11±0.06 | 0.13±0.08 | 0.12±0.07 | 0.09±0.07 | 0.11±0.06 | 0.1±0.05 |
| C14:1-OH | 0.02±0.01 | 0.02±0.01 | 0.02±0.01 | 0.02±0.01 | 0.02±0.01 | 0.01±0.01 |
| C14:2 | 0.02±0.02 | 0.03±0.02 | 0.02±0.02 | 0.02±0.02 | 0.02±0.01 | 0.02±0.01 |
| C14:2-OH | 0.01±0.01 | 0.01±0.01 | 0.01±0.01 | 0.01±0.01 | 0.01±0.01 | 0.01±0.01 |
| C16 | 0.24±0.19 | 0.36±0.28 | 0.26±0.22 | 0.21±0.21 | 0.25±0.2 | 0.19±0.15 |
| C16-OH | 0.01±0.01 | 0.02±0.01 | 0.01±0.01 | 0.01±0.01 | 0.01±0.01 | 0.01±0.01 |
| C16:1 | 0.09±0.07 | 0.12±0.09 | 0.11±0.08 | 0.08±0.08 | 0.11±0.08 | 0.08±0.06 |
| C16:1-OH | 0.01±0.01 | 0.02±0.01 | 0.02±0.01 | 0.01±0.01 | 0.02±0.01 | 0.01±0.01 |
| C16-2 | 0.02±0.02 | 0.03±0.02 | 0.02±0.02 | 0.02±0.02 | 0.02±0.01 | 0.01±0.01 |
| C16:2-OH | 0.01±0.01 | 0.01±0.01 | 0.01±0.01 | 0.01±0.01 | 0.01±0.01 | 0.01±0.01 |
| C18 | 0.05±0.03 | 0.08±0.04 | 0.05±0.03 | 0.05±0.04 | 0.06±0.04 | 0.05±0.02 |
| C18:1 | 0.19±0.18 | 0.28±0.23 | 0.19±0.16 | 0.18±0.22 | 0.22±0.19 | 0.15±0.14 |
| C18:1-OH | 0.02±0.02 | 0.03±0.02 | 0.02±0.02 | 0.02±0.02 | 0.02±0.02 | 0.02±0.01 |
| C18:2 | 0.07±0.08 | 0.11±0.1 | 0.08±0.07 | 0.07±0.08 | 0.08±0.06 | 0.06±0.05 |
| C2 | 19.3±5.16 | 20.36±6.17 | 20.69±6.1 | 17.93±5.51 | 20.06±6.22 | 17.93±3.64 |
| C3 | 1.04±0.69 | 0.8±0.68 | 0.82±0.44 | 1.13±0.83 | 1.26±0.96 | 1.16±0.73 |
| C3-DC (C4-OH) | 0.26±0.11 | 0.27±0.1 | 0.28±0.15 | 0.21±0.11 | 0.27±0.18 | 0.21±0.08 |
| C3-OH | 0.03±0.01 | 0.02±0.01 | 0.02±0.01 | 0.03±0.02 | 0.02±0.01 | 0.03±0.01 |
| C3:1 | 0.01±0.01 | 0.01±0.01 | 0.01±0.01 | 0.01±0.01 | 0.01±0.01 | 0.01±0.01 |
| C4 | 1.36±1.4 | 0.7±0.38 | 1.02±0.83 | 1.54±2.56 | 1.26±0.95 | 1.22±0.94 |
| C4:1 | 0.02±0.01 | 0.02±0.01 | 0.02±0.01 | 0.02±0.01 | 0.02±0.01 | 0.02±0.01 |
| C4:1-DC (C6) | 0.16±0.13 | 0.13±0.08 | 0.15±0.11 | 0.21±0.33 | 0.18±0.13 | 0.14±0.09 |
| C6:1 | 0.02±0 | 0.01±0.01 | 0.02±0.01 | 0.02±0.01 | 0.02±0.01 | 0.02±0.01 |
| C7-DC | 0.02±0.01 | 0.02±0 | 0.02±0.01 | 0.02±0.01 | 0.02±0.01 | 0.02±0.01 |
| C8 | 0.14±0.05 | 0.11±0.04 | 0.13±0.04 | 0.12±0.04 | 0.13±0.06 | 0.14±0.06 |
| C8:1 | 0.02±0.01 | 0.02±0.01 | 0.02±0.01 | 0.02±0.01 | 0.02±0.01 | 0.02±0.01 |
| C9 | 0.02±0.01 | 0.02±0.01 | 0.02±0.01 | 0.02±0.01 | 0.02±0.01 | 0.02±0.01 |
| **Amino acids** | | | | | | |
| Arg | 115.44±46.25 | 87.07±49.75 | 117.95±47.63 | 117.23±40.61 | 139.08±61.98 | 138.39±43.99 |
| Gln | 628.28±168.55 | 524.54±234.83 | 585.77±178.89 | 620.21±186.03 | 603.41±198.32 | 643.06±187.89 |
| Gly | 304.6±76.5 | 244.69±70.25 | 293.46±68.74 | 300.32±78.67 | 294.41±79.48 | 296.83±63.39 |
| His | 79.67±23.66 | 67.5±23.41 | 73.21±23.3 | 75.81±22.77 | 77.81±21.96 | 75.24±14.06 |
| Met | 59.51±21.79 | 58.56±22.1 | 55.52±18.41 | 68.91±21.09 | 71.84±21.51 | 65.06±17.74 |
| Orn | 106.07±42.6 | 107.03±42.06 | 83.15±25.35 | 118±48.56 | 108.23±40.1 | 94.93±28.51 |
| Phe | 78.21±21.67 | 70.28±24.21 | 73.63±24.43 | 79.1±21.48 | 81.84±23.38 | 78.96±14.84 |
| Pro | 124.36±63.14 | 109.41±66.22 | 103.21±44.8 | 134.38±58.54 | 127.79±56.96 | 122.56±47.8 |
| Ser | 133.46±41.32 | 113.89±43.88 | 120.28±40.47 | 155.36±49.41 | 158.16±55.31 | 149.71±41.61 |
| Thr | 121.83±40.07 | 118.9±39.39 | 117.93±31.08 | 154.88±50.03 | 163.15±56.16 | 143.19±40.17 |
| Trp | 80.63±17.32 | 81.1±21.67 | 92.54±26.14 | 110.44±20.08 | 114.16±28.35 | 120.96±26.97 |
| Tyr | 104.42±49.78 | 101.38±46.7 | 102.26±42.61 | 112.52±46.24 | 117.16±44.68 | 118.11±50.33 |
| Val | 348.16±86.23 | 353.08±123.64 | 329.46±92.39 | 385.96±120.43 | 377.06±89.27 | 356.94±66.19 |
| xLeu | 356.44±91.4 | 352.85±129.19 | 341.46±94.57 | 365.39±102.54 | 393.35±103.54 | 351.56±68.04 |
| **Lyso-phosphatidyl-colines** | | | | | | |
| lysoPC a C14:0 | 3.25±1.14 | 2.21±0.68 | 3.64±1.54 | 3.29±1.28 | 3.67±1.55 | 3.99±1.89 |
| lysoPC a C16:0 | 281.36±62.2 | 221.23±36.74 | 266±77.9 | 211.86±45.7 | 234.29±38.07 | 235.17±50.78 |
| lysoPC a C16:1 | 8.26±2.18 | 5.61±1.34 | 8.48±2.06 | 4.75±2.05 | 5.26±1.72 | 5.65±2.13 |
| lysoPC a C17:0 | 3.61±0.98 | 2.65±0.9 | 3.42±1.06 | 3.11±0.61 | 3.14±0.75 | 3.49±1 |
| lyasoPC a C18:0 | 109.7±32.54 | 84.19±21.16 | 100.83±31.81 | 106.94±24.68 | 119.66±29.9 | 119.01±28.78 |
| lysoPC a C18:1 | 59.17±17.86 | 39.18±9.35 | 62.31±23.75 | 41.93±12.2 | 47.11±13.58 | 53.74±20.78 |
| lysoPC a C18:2 | 135.3±45.07 | 93.64±24.09 | 146.65±63.68 | 105.77±23.23 | 121.99±38.67 | 135.33±50.32 |
| lysoPC a C20:3 | 11.46±5.87 | 5.48±2.7^a^ | 12.43±8.62 | 6.35±2.89 | 7.62±3.83 | 10±5.56 |
| lysoPC a C20:4 | 41.26±16.48 | 30.71±7.4 | 48.88±17.28 | 39.45±10.87 | 49.94±16.3 | 62.17±23.32 |
| lysoPC a C24:0 | 1.18±0.35 | 1.12±0.23 | 1.37±0.52 | 0.86±0.25 | 1.02±0.36 | 0.98±0.26 |
| lysoPC a C26:0 | 1.52±0.71 | 1.43±0.44 | 1.96±1.08 | 0.92±0.31 | 1.14±0.56 | 1.14±0.37 |
| lysoPC a C26:1 | 2.56±0.34 | 2.49±0.19 | 2.75±0.44 | 2.55±0.2 | 2.62±0.29 | 2.66±0.35 |
| lysoPC a C28:0 | 0.85±0.34 | 0.78±0.23 | 1.06±0.53 | 0.59±0.24 | 0.72±0.38 | 0.71±0.25 |
| lysoPC a C28:1 | 0.77±0.35 | 0.73±0.19 | 0.97±0.48 | 0.51±0.2 | 0.61±0.27 | 0.63±0.23 |
| lysoPC a C6:0 | 0.03±0.01 | 0.03±0.01 | 0.03±0.03 | 0.02±0.01 | 0.02±0.01 | 0.03±0.01 |
| **Diacyl-phosphatidyl-cholines** | | | | | | |
| PC aa C24:0 | 0.33±0.17 | 0.34±0.11 | 0.42±0.21 | 0.23±0.1 | 0.31±0.16 | 0.29±0.1 |
| PC aa C26:0 | 1.79±0.9 | 1.66±0.61 | 2±1.21 | 1.09±0.42 | 1.21±0.6 | 1.22±0.38 |
| PC aa C28:1 | 0.47±0.25 | 0.44±0.17 | 0.6±0.38 | 0.32±0.13 | 0.37±0.2 | 0.38±0.12 |
| PC aa C30:0 | 1.15±0.18 | 1.04±0.24 | 1.17±0.25 | 0.93±0.17 | 1.07±0.28 | 1.06±0.22 |
| PC aa C30:2 | 1.24±0.22 | 1.14±0.16 | 1.22±0.35 | 0.8±0.17 | 0.92±0.19 | 0.92±0.2 |
| PC aa C32:0 | 15.64±3.65 | 14.2±3.9 | 16.22±2.92 | 11.43±2.26 | 14.23±3.65 | 13.72±3.29 |
| PC aa C32:1 | 9.39±3.67 | 7.25±2.38 | 8.81±3.35 | 4.87±1.64 | 6.21±2.26 | 5.78±1.63 |
| PC aa C32:2 | 1.93±0.42 | 1.71±0.47 | 1.85±0.43 | 1.1±0.44 | 1.27±0.4 | 1.22±0.42 |
| PC aa C32:3 | 0.23±0.06 | 0.2±0.04 | 0.22±0.07 | 0.15±0.05 | 0.18±0.05 | 0.18±0.06 |
| PC aa C34:1 | 212.84±60.23 | 165.15±41.7 | 184.38±59.84 | 101.35±29.25 | 124.78±38.84 | 116.78±27.98 |
| PC aa C34:2 | 375.72±64.19 | 310.08±61.11 | 345.54±72.47 | 229.93±41.68 | 263.24±53.04 | 257.94±56.53 |
| PC aa C34:3 | 20.19±5.17 | 19.07±6.3 | 19.43±6.4 | 8.41±4.19 | 10.14±4.58 | 8.9±4.71 |
| PC aa C34:4 | 0.63±0.18 | 0.61±0.2 | 0.65±0.2 | 0.3±0.15 | 0.36±0.15 | 0.35±0.15 |
| PC aa C36:0 | 6.38±1.74 | 5.04±1.08 | 5.63±1.54 | 3.81±0.83 | 4.72±1.54 | 4.45±1.02 |
| PC aa C36:1 | 55.34±16.36 | 40.28±9.52 | 45.86±15.35 | 31.18±7.32 | 37.21±12.38 | 35.39±8.34 |
| PC aa C36:2 | 241.92±62.78 | 182.92±43.92^a^ | 211.54±61.84 | 153.13±29.38 | 178.65±49.72 | 169.62±43.56 |
| PC aa C36:3 | 124.48±44.81 | 87.39±27.52 | 111.82±34.36 | 62.47±17.11 | 71.06±22.89 | 70.58±17.83 |
| PC aa C36:4 | 174.28±42.83 | 157.38±31.71 | 164.85±29.14 | 105.17±29.3 | 132.71±34.86 | 127.95±31.2 |
| PC aa C36:5 | 10.29±3.49 | 7.87±1.78 | 9.28±3.07 | 4.61±1.41 | 5.71±2.1 | 5.3±1.41 |
| PC aa C36:6 | 0.55±0.13 | 0.46±0.1 | 0.5±0.13 | 0.26±0.09 | 0.3±0.11 | 0.3±0.09 |
| PC aa C38:0 | 2.42±0.62 | 2.06±0.36 | 2.25±0.45 | 1.7±0.42 | 1.97±0.61 | 1.88±0.43 |
| PC aa C38:1 | 6.99±1.66 | 5.39±1.16^a^ | 5.67±1.54 | 3.56±0.73 | 4.18±1.4 | 4.05±0.85 |
| PC aa C38:3 | 38.32±14.93 | 25.74±6.97 | 33.05±12.48 | 22.14±5.39 | 27.3±9.54 | 26.87±5.86 |
| PC aa C38:4 | 101.37±30.05 | 93.11±12.29 | 95.7±18.75 | 82.45±20.67 | 106.89±26.83 | 102.78±24.54 |
| PC aa C38:5 | 56.2±16.88 | 45.48±8.84 | 53.82±11.1 | 29.65±8.57 | 36.25±10.84 | 36.07±8.03 |
| PC aa C38:6 | 139.58±37.22 | 121.21±21.48 | 122.72±30.81 | 69.26±24.91 | 89.51±27.13 | 82.21±23.98 |
| PC aa C40:1 | 0.65±0.17 | 0.56±0.09 | 0.64±0.15 | 0.43±0.08 | 0.5±0.13 | 0.46±0.08 |
| PC aa C40:2 | 0.91±0.36 | 0.68±0.21 | 0.76±0.3 | 0.42±0.14 | 0.55±0.3 | 0.46±0.16 |
| PC aa C40:3 | 1.66±0.76 | 1.06±0.46 | 1.38±0.73 | 0.49±0.12 | 0.6±0.21 | 0.53±0.11 |
| PC aa C40:4 | 4.74±1.94 | 3.35±1.15 | 4.1±2.09 | 1.63±0.39 | 2.01±0.58 | 1.89±0.34 |
| PC aa C40:5 | 12.78±3.97 | 9.96±1.47 | 11.18±4.4 | 6.88±2.06 | 8.71±2.42 | 8.03±1.66 |
| PC aa C40:6 | 48.13±15.14 | 39.85±5.83 | 40.49±12.91 | 32.24±10.34 | 41.64±10.52 | 37.61±9.18 |
| PC aa C42:0 | 0.25±0.06 | 0.24±0.04 | 0.25±0.07 | 0.22±0.06 | 0.28±0.11 | 0.25±0.07 |
| PC aa C42:1 | 0.29±0.07 | 0.25±0.05 | 0.28±0.06 | 0.2±0.04 | 0.25±0.08 | 0.23±0.06 |
| PC aa C42:2 | 0.44±0.13 | 0.38±0.09 | 0.42±0.12 | 0.21±0.06 | 0.29±0.11 | 0.25±0.07 |
| PC aa C42:4 | 0.51±0.18 | 0.41±0.1 | 0.44±0.19 | 0.24±0.07 | 0.29±0.1 | 0.25±0.06 |
| PC aa C42:5 | 0.88±0.36 | 0.6±0.25 | 0.73±0.4 | 0.28±0.06 | 0.33±0.09 | 0.31±0.05 |
| PC aa C42:6 | 1.96±0.83 | 1.3±0.62 | 1.55±0.92 | 0.53±0.08 | 0.58±0.13 | 0.59±0.07 |
| **Acyl-alkyl-phosphatidyl-cholines** | | | | | | |
| PC ae C30:0 | 0.26±0.09 | 0.24±0.05 | 0.29±0.1 | 0.17±0.04 | 0.19±0.06 | 0.19±0.04 |
| PC ae C30:1 | 0.65±0.22 | 0.53±0.15 | 0.68±0.25 | 0.54±0.13 | 0.56±0.17 | 0.6±0.13 |
| PC ae C30:2 | 0.14±0.06 | 0.13±0.03 | 0.16±0.07 | 0.08±0.03 | 0.11±0.05 | 0.1±0.03 |
| PC ae C32:1 | 0.94±0.19 | 0.84±0.13 | 0.94±0.21 | 0.69±0.14 | 0.8±0.2 | 0.82±0.15 |
| PC ae C32:2 | 0.39±0.1 | 0.33±0.07 | 0.4±0.13 | 0.21±0.05 | 0.24±0.08 | 0.26±0.05 |
| PC ae C34:0 | 0.9±0.16 | 0.75±0.15 | 0.87±0.15 | 0.63±0.12 | 0.74±0.21 | 0.74±0.15 |
| PC ae C34:1 | 4.64±0.99 | 3.71±0.73^a^ | 4.11±0.97 | 2.75±0.59 | 3.14±0.85 | 3.21±0.64 |
| PC ae C34:2 | 4.85±0.95 | 3.83±0.88 | 4±0.99 | 2.45±0.47 | 2.66±0.71 | 2.73±0.57 |
| PC ae C34:3 | 1.27±0.32 | 0.97±0.35 | 1.07±0.36 | 0.72±0.14 | 0.81±0.3 | 0.81±0.19 |
| PC ae C36:0 | 1.1±0.26 | 1±0.24 | 1.03±0.28 | 0.71±0.24 | 0.83±0.3 | 0.74±0.16 |
| PC ae C36:1 | 6.01±1.77 | 4.9±1.03 | 5.28±1.42 | 3.31±1.14 | 3.55±1.2 | 3.34±0.76 |
| PC ae C36:2 | 11.66±2.83 | 9.17±2.81 | 10.52±2.79 | 6.5±1.47 | 7.28±2.57 | 7.34±2.15 |
| PC ae C36:3 | 2.62±0.49 | 2.1±0.48^a^ | 2.35±0.47 | 1.45±0.32 | 1.52±0.41 | 1.64±0.38 |
| PC ae C36:4 | 4.73±0.95 | 4.55±0.74 | 4.71±0.79 | 3.43±0.86 | 3.84±0.81 | 4.11±0.98 |
| PC ae C36:5 | 2.25±0.52 | 1.97±0.39 | 2.15±0.41 | 1.99±0.54 | 2.29±0.44 | 2.39±0.56 |
| PC ae C38:0 | 4.32±1.31 | 3.34±0.63 | 3.71±0.78 | 1.9±0.55 | 2.15±0.67 | 2.08±0.48 |
| PC ae C38:1 | 3.89±1.03 | 3.09±0.64 | 3.29±0.73 | 2.92±0.85 | 3.14±1.1 | 2.99±0.65 |
| PC ae C38:2 | 8.38±2.5 | 6.37±2.23 | 7.39±2.43 | 3.9±0.97 | 4.51±1.7 | 4.24±1.26 |
| PC ae C38:3 | 3.55±1.43 | 2.61±0.65 | 3.03±0.71 | 2.16±0.69 | 2.27±0.85 | 2.21±0.59 |
| PC ae C38:4 | 4.94±1.26 | 4.55±0.84 | 4.75±0.8 | 3.73±0.87 | 4.34±0.94 | 4.55±1.02 |
| PC ae C38:5 | 3.46±0.75 | 3.18±0.44 | 3.5±0.64 | 2.6±0.65 | 2.93±0.56 | 3.06±0.74 |
| PC ae C38:6 | 2.92±0.57 | 2.68±0.36 | 2.74±0.6 | 1.93±0.5 | 2.18±0.43 | 2.24±0.52 |
| PC ae C40:0 | 20.2±4.56 | 16.48±3.16 | 19.29±3.64 | 12.46±2.51 | 13.84±2.85 | 13.96±2.31 |
| PC ae C40:1 | 3.34±0.95 | 2.66±0.69 | 2.99±0.83 | 1.71±0.3 | 2.07±0.56 | 1.89±0.35 |
| PC ae C40:2 | 1.22±0.45 | 1.02±0.22 | 1.13±0.28 | 0.77±0.23 | 0.93±0.35 | 0.82±0.19 |
| PC ae C40:3 | 1.58±0.74 | 1.18±0.28 | 1.29±0.37 | 1.18±0.69 | 1.13±0.52 | 1.04±0.34 |
| PC ae C40:4 | 2.6±0.84 | 2.23±0.38 | 2.32±0.46 | 1.55±0.37 | 1.78±0.49 | 1.75±0.43 |
| PC ae C40:5 | 2.55±1.24 | 1.87±0.42 | 2.01±0.46 | 1.55±0.58 | 1.5±0.45 | 1.48±0.34 |
| PC ae C40:6 | 3.31±0.85 | 2.81±0.37 | 2.94±0.61 | 2.2±0.64 | 2.54±0.51 | 2.55±0.56 |
| PC ae C42:0 | 1.39±0.45 | 1.03±0.34 | 1.26±0.41 | 0.57±0.11 | 0.64±0.17 | 0.65±0.1 |
| PC ae C42:1 | 0.85±0.29 | 0.67±0.19 | 0.82±0.22 | 0.48±0.13 | 0.58±0.25 | 0.54±0.11 |
| PC ae C42:2 | 0.8±0.28 | 0.66±0.18 | 0.78±0.23 | 0.43±0.13 | 0.56±0.25 | 0.5±0.11 |
| PC ae C42:3 | 1.17±0.37 | 0.93±0.22 | 1.07±0.3 | 0.56±0.12 | 0.7±0.22 | 0.65±0.11 |
| PC ae C42:4 | 0.59±0.21 | 0.5±0.1 | 0.54±0.15 | 0.34±0.1 | 0.42±0.15 | 0.36±0.1 |
| PC ae C42:5 | 1.08±0.39 | 0.83±0.12^a^ | 0.88±0.16 | 0.82±0.32 | 0.81±0.22 | 0.75±0.16 |
| PC ae C44:3 | 0.23±0.08 | 0.2±0.04 | 0.22±0.07 | 0.14±0.04 | 0.18±0.07 | 0.15±0.04 |
| PC ae C44:4 | 0.24±0.07 | 0.2±0.04 | 0.23±0.07 | 0.14±0.03 | 0.18±0.07 | 0.16±0.04 |
| PC ae C44:5 | 0.25±0.07 | 0.21±0.04 | 0.24±0.05 | 0.15±0.03 | 0.17±0.05 | 0.17±0.03 |
| PC ae C44:6 | 0.25±0.05 | 0.22±0.03 | 0.24±0.05 | 0.17±0.03 | 0.21±0.06 | 0.19±0.04 |
| **Hydroxy-sphingomyelins** | | | | | | |
| SM(OH)C14:1 | 1.19±0.29 | 0.96±0.24 | 1.15±0.21 | 1.4±0.29 | 1.42±0.39 | 1.56±0.37 |
| SM(OH)C16:1 | 0.39±0.07 | 0.35±0.05 | 0.41±0.07 | 0.46±0.1 | 0.47±0.09 | 0.52±0.1 |
| SM(OH)C22:1 | 5.75±1.19 | 4.72±1.2 | 5.06±1.13 | 4.38±0.87 | 4.76±1.39 | 4.96±1.03 |
| SM(OH)C22:2 | 2.87±0.68 | 2.33±0.4 | 2.6±0.42 | 2.21±0.44 | 2.32±0.67 | 2.42±0.52 |
| SM(OH)C24:1 | 0.67±0.15 | 0.57±0.11 | 0.64±0.11 | 0.61±0.11 | 0.69±0.2 | 0.68±0.14 |
| **Sphingomyelins** | | | | | | |
| SM C16:0 | 20.31±4.41 | 18±2.82 | 18.84±2.49 | 22.91±4.57 | 24.69±4.84 | 25.88±4.29 |
| SM C16:1 | 3.69±0.56 | 3.67±0.43 | 3.57±0.91 | 2.52±0.53 | 2.79±0.46 | 2.89±0.62 |
| SM C18:0 | 2.19±0.52 | 2.25±0.47 | 2.31±0.54 | 4.5±1.62 | 4.99±1.36 | 5.15±1.23 |
| SM C18:1 | 0.71±0.17 | 0.73±0.14 | 0.74±0.18 | 1.14±0.36 | 1.27±0.28 | 1.31±0.33 |
| SM C20:2 | 0.29±0.09 | 0.29±0.09 | 0.31±0.1 | 0.2±0.08 | 0.23±0.06 | 0.24±0.07 |
| SM C20:3 | 4.26±1.28 | 3.43±0.66 | 4.02±1.13 | 2.14±0.63 | 2.51±0.86 | 2.44±0.6 |
| SM C24:0 | 12.3±2.46 | 9.88±1.63 | 9.91±2.19 | 7.06±1.47 | 7.93±2.29 | 8.22±1.54 |
| SM C24:1 | 28.41±7.03 | 21.65±3.78 | 25.3±5.81 | 21.67±5.03 | 24.24±6.92 | 26.07±4.86 |
| SM C26:0 | 0.4±0.14 | 0.34±0.08 | 0.37±0.11 | 0.25±0.08 | 0.3±0.17 | 0.27±0.09 |
| SM C26:1 | 0.58±0.23 | 0.4±0.15 | 0.5±0.23 | 0.23±0.05 | 0.27±0.09 | 0.26±0.06 |
| **Hexoses** | | | | | | |
| H | 10462.08±4387.31 | 8477.38±5533.04 | 10054.69±6835.55 | 8303.18±3210.57 | 9564±5260.55 | 8790.28±4042.01 |

F:WT; M:WT: wildtype offspring of wildtype fathers and wildtype mothers; F:WT;M:eNOS+/-: wildtype offspring of wildtype fathers and eNOS heterozygous mothers; F:eNOS+/-;M:WT: wildtype offspring of eNOS heterozygous fathers and wildtype mothers. PC = phosphatidylcholine; a = acyl; LPC = lysophosphatidylcholine; aa = diacyl; ae = acyl-alkyl; SM = Sphingomyelins; OH = hydroxy. Data are given as mean ± SEM. a: p<0.05, F:WT; M:eNOS+/- vs F:WT; M:WT.

**Supplementary Table 4. Central carbon metabolites concentration (****nmol/mg protein) in liver tissue**

| Variable | Male offspring | | | Female offspring | | |
| --- | --- | --- | --- | --- | --- | --- |
|  | F: WT; M: WT  (n=21) | F: WT; M: eNOS+/-(n=16) | F: eNOS+/-; M: WT (n=9) | F: WT; M: WT (n=27) | F: WT; M: eNOS+/-(n=17) | F: eNOS+/-; M: WT (n=15) |
| Ribose 5-phosphate | 1.12 ± 0.06 | 1.25 ± 0.06 | 1.24±0.09 | 1.55 ± 0.10 | 1.56 ± 0.09 | 1.59±0.07 |
| Glucose 6-phosphate | 2.48 ± 0.17 | 1.58±0.22^a^ | 2.11±0.23 | 2.26 ± 0.20 | 1.61±0.20^a^ | 1.91±0.27 |
| Fructose 6-phosphate | 1.04 ± 0.08 | 0.67 ± 0.09^a^ | 0.72±0.08 | 0.94 ± 0.08 | 0.67 ± 0.08^a^ | 0.70±0.09 |
| Phosphoenol-pyruvate | 0.62 ± 0.04 | 0.55 ± 0.04 | 0.52±0.04 | 0.61 ± 0.04 | 0.51 ± 0.05 | 0.53±0.05 |
| 6-Phospho-gluconate | 0.50 ± 0.05 | 0.52 ± 0.05 | 0.67±0.04 | 0.60 ± 0.04 | 0.44 ± 0.06 | 0.57±0.07 |
| Fructose 1,6- bisphosphate | 0.18 ± 0.02 | 0.12 ± 0.02^a^ | 0.14±0.02 | 0.20 ± 0.02 | 0.12 ± 0.01^a^ | 0.18±0.02 |
| Seduheptulose 7-phoshate | 1.96 ± 0.14 | 1.78 ± 0.17 | 2.09±0.33 | 1.99 ± 0.14 | 1.91 ± 0.22 | 2.20±0.28 |
| Fumarate | 4.07 ± 0.27 | 2.52±0.19^a^ | 2.53±0.16^b^ | 4.43 ± 0.30 | 2.66 ± 0.19 ^a^ | 2.29±0.13^b^ |
| Hydroxy-butyrate | 1.54 ± 0.12 | 1.97+0.40 | 1.66±0.13 | 2.10 ± 0.16 | 2.35±0.35 | 1.68±0.06 |
| Citrate | 0.58 ± 0.08 | 0.56+0.05 | 0.54±0.04 | 0.72 ± 0.08 | 0.66±0.10 | 0.54±0.04 |
| Malate | 9.73 ± 0.55 | 8.59+0.43 | 9.92±0.47 | 9.21 ± 0.51 | 8.82±0.48 | 9.21±0.56 |
| 2- / 3-Phospho-glycerate | 5.21 ± 0.33 | 4.99 ± 0.42 | 4.60±0.41 | 5.02 ± 0.26 | 5.04 ± 0.48 | 4.58±0.42 |
| Ribulose 5-phosphate / Xylulose 5-phosphate | 5.45 ± 0.72 | 5.76 ± 0.70 | 6.80±1.13 | 6.01 ± 0.68 | 6.34 ± 0.75 | 7.93±0.94 |

F:WT; M:WT: wildtype offspring of wildtype fathers and wildtype mothers; F:WT;M:eNOS+/-: wildtype offspring of wildtype fathers and eNOS heterozygous mothers; F:eNOS+/-;M:WT: wildtype offspring of eNOS heterozygous fathers and wildtype mothers. Data are given as mean ± SEM. a: p<0.05, F:WT; M:eNOS+/- vs F:WT; M:WT; b: p<0.05, F:eNOS+/-; M:WT vs F:WT; M:WT.

**Supplementary Table 5. Correlation analysis between AUC of plasma glucose and insulin, fat content, glycogen, and metabolites in wild type offspring with eNOS+/- mothers.**

| Variable | Male offspring | | | | Female offspring | | | |
| --- | --- | --- | --- | --- | --- | --- | --- | --- |
|  | AUC of plasma glucose | AUC of plasma insulin | Fat content | Glycogen | AUC of plasma glucose | AUC of plasma insulin | Fat Content | Glycogen |
| Glucose 6-phosphate | 0.103 | -0.028 | -.401* | 0.19 | -0.098 | 0.129 | 0.047 | .393* |
| Fructose 6-phosphate | 0.136 | -0.008 | 0.119 | 0.111 | -0.075 | 0.13 | 0.012 | .388* |
| Fumarate | -.407* | 0.285 | -0.246 | -0.193 | -.431** | -0.215 | -.338* | 0.037 |
| Fructose 1,6- bisphosphate | -0.013 | 0.073 | -0.178 | -0.059 | -0.048 | 0.087 | -0.069 | .404* |
| lysoPC a C20:3 | 0.016 | 0.036 | 0.158 | -0.242 | 0.205 | .448* | 0.22 | 0.03 |
| PC aa C36:2 | 0.039 | -0.007 | 0.318 | -0.152 | 0.143 | 0.161 | 0.25 | -0.049 |
| PC aa C38:1 | -0.177 | -0.04 | 0.242 | -0.177 | 0.218 | 0.357 | 0.183 | -0.044 |
| PC ae C34:1 | 0.086 | 0.01 | 0.316 | -0.163 | 0.299 | 0.355 | 0.218 | 0.164 |
| PC ae C36:3 | 0.142 | 0.004 | 0.064 | -0.284 | 0.285 | 0.235 | 0.206 | 0.188 |
| PC ae C42:5 | -0.128 | 0.064 | .368* | -0.105 | -0.24 | -0.146 | -0.111 | 0.001 |

AUC: area under curve. *: p<0.05, **: p<0.01.

**Supplementary Table 6. Correlation analysis between AUC of plasma glucose and insulin, fat content, glycogen, and metabolites in wild type offspring with eNOS+/- fathers.**

| Variable | Male offspring | | | | Female offspring | | | |
| --- | --- | --- | --- | --- | --- | --- | --- | --- |
|  | AUC of plasma glucose | AUC of plasma insulin | Fat content | Glycogen | AUC of plasma glucose | AUC of plasma insulin | Fat Content | Glycogen |
| Glucose 6-phosphate | 0.239 | -0.037 | 0.085 | 0.076 | 0.079 | 0.224 | 0.191 | -0.12 |
| Fructose 6-phosphate | 0.23 | -0.172 | 0.03 | -0.098 | 0.14 | 0.26 | 0.068 | -0.169 |
| Fumarate | -.374* | -0.118 | -0.318 | -0.396* | -0.297 | -0.332 | -0.28 | -.426* |
| Fructose 1,6- bisphosphate | 0.033 | -0.013 | -0.176 | -0.09 | 0.161 | 0.008 | -0.032 | 0.206 |
| lysoPC a C20:3 | 0.118 | 0.335 | .394* | 0.059 | 0.149 | .460** | -0.052 | .375* |
| PC aa C36:2 | 0.112 | 0.166 | 0.318 | -0.165 | 0.066 | 0.322 | 0.089 | 0.206 |
| PC aa C38:1 | -0.218 | -0.12 | 0.113 | -.459* | 0.072 | .404* | 0.138 | 0.135 |
| PC ae C34:1 | 0.097 | 0.156 | 0.216 | -0.238 | 0.22 | .409* | 0.053 | 0.252 |
| PC ae C36:3 | 0.171 | -0.028 | -0.046 | -0.324 | 0.26 | 0.291 | -0.043 | 0.144 |
| PC ae C42:5 | -0.124 | -0.013 | 0.155 | -0.302 | -.361 | -0.241 | -0.195 | -0.191 |

AUC: area under curve. *: p<0.05, **: p<0.01.

**Supplementary Table 7. Complete real time PCR results.**

| Variable | Male offspring | | | Female offspring | | |
| --- | --- | --- | --- | --- | --- | --- |
|  | F: WT; M: WT  (n=22) | F: WT; M: eNOS+/-(n=9) | F: eNOS+/-; M: WT (n=10) | F: WT; M: WT (n=25) | F: WT; M: eNOS+/-(n=13) | F: eNOS+/-; M: WT (n=10) |
| **NO-synthase expression** | | | | | | |
| eNOS (Nos3) | 1.00 ± 0.07 | 1.10 ± 0.14 | 1.06 ± 0.07 | 1.00 ± 0.11 | 0.83 ± 0.12 | 1.00 ± 0.16 |
| iNOS (Nos2) | 1.00 ± 0.19 | 0.84 ± 0.13 | 0.84 ± 0.11 | 1.00 ± 0.19 | 1.57 ± 0.75 | 0.95 ± 0.24 |
| **Genes involved in regulation of metabolic process, energy homeostasis and fat storage** | | | | | | |
| Chrebp (Mlxipl) | 1.00 ± 0.07 | 0.95 ± 0.09 | 1.32±0.05^b^ | 1.00 ± 0.08 | 0.89 ± 0.08 | 1.03±0.16^c^ |
| Srebf1c | 1.00 ± 0.21 | 0.98 ± 0.15 | 0.91±0.12 | 1.00 ± 0.11 | 0.49 ± 0.05^a^ | 0.86±0.17^c^ |
| PPARα | 1.00 ± 0.07 | 0.79 ± 0.08 | 1.13±0.10^c^ | 1.00 ± 0.08 | 0.96 ± 0.13 | 1.47±0.19^bc^ |
| PPARγ | 1.00 ± 0.10 | 0.56 ± 0.05^a^ | 1.09±0.11 | 1.00 ± 0.10 | 1.04 ± 0.14 | 1.52±0.20^bc^ |
| PPARγ Co1α | 1+0.29 | 0.46+0.11 | 1.00+0.25 | 1+0.17 | 1.15+0.2 | 1.32+0.19 |
| GR (Nr3c1) | 1.00 ± 0.10 | 1.41± 0.19 | 1.82±0.16^b^ | 1.00 ± 0.13 | 0.68 ± 0.06 | 1.40±0.30^c^ |
| Tfam | 1.00 ± 0.08 | 0.99 ± 0.14 | 1.57±0.05^bc^ | 1.00 ± 0.08 | 1.20 ± 0.16 | 1.12±0.11 |
| Tfb1m | 1.00 ± 0.05 | 1.06 ± 0.14 | 1.00±0.05 | 1.00 ± 0.05 | 1.03 ± 0.10 | 0.87±0.04 |
| Tfb2m | 1.00 ± 0.09 | 1.02 ± 0.17 | 1.92±0.06^bc^ | 1.00 ± 0.12 | 1.16 ± 0.16 | 1.38±0.22 |
| Nrf1 | 1.00 ± 0.05 | 1.15 ± 0.15 | 1.20±0.05 | 1.00 ± 0.04 | 1.00 ± 0.07 | 1.04±0.08 |
| Nampt | 1.00 ± 0.12 | 1.16 ± 0.21 | 1.68±0.22^bc^ | 1.00 ± 0.11 | 0.84 ± 0.12 | 1.00±0.12 |
| AMPK (Prkaa2) | 1.00 ± 0.15 | 1.32 ± 0.28 | 1.16±0.15 | 1.00 ± 0.10 | 0.73 ± 0.08 | 1.30±0.23^c^ |
| Fbp1 | 1.00 ± 0.10 | 1.32 ± 0.15 | 1.24±0.17 | 1.00 ± 0.11 | 0.86 ± 0.08 | 1.14±0.17 |
| Gck | 1.00 ± 0.10 | 1.35 ± 0.39 | 0.99±0.18 | 1.00 ± 0.16 | 0.57 ± 0.08^a^ | 1.11±0.16^c^ |
| Pepck (Pck1) | 1.00 ± 0.07 | 1.41 ± 0.22^a^ | 1.41±0.29^b^ | 1.00 ± 0.11 | 0.72 ± 0.05 | 1.1±0.27 |
| PK-L (Pklr) | 1.00+0.1 | 0.98+0.08 | 0.94+0.08 | 1.00+0.06 | 1.18+0.09 | 1.13+0.20 |
| G6Pase (G6pc) | 1.00 ± 0.15 | 1.07± 0.15 | 1.54±0.11^b^ | 1.00 ± 0.17 | 1.21 ± 0.19 | 1.14±0.16 |
| Glut2 (Slc2a2) | 1.00 ± 0.09 | 1.22 ± 0.17 | 2.07±0.13^bc^ | 1.00 ± 0.13 | 0.77 ± 0.14 | 1.38±0.20^c^ |
| FAS (Fasn) | 1.00 ± 0.27 | 0.60± 0.09 | 0.77±0.11 | 1.00 ± 0.08 | 1.00 ± 0.17 | 1.62±0.33^bc^ |
| Acc1 (Acaca) | 1+0.12 | 0.83+0.11 | 1.68+0.15 | 1+0.09 | 1.03±0.13 | 1.27+0.20 |
| Cpt1a | 1.00 ± 0.12 | 1.10 ± 0.15 | 0.95±0.1 | 1.00 ± 0.12 | 0.86 ± 0.10 | 1.30±0.18^c^ |
| HSL (Lipe) | 1.00 ± 0.10 | 0.92 ± 0.11 | 0.90±0.10 | 1.00 ± 0.12 | 0.87 ± 0.16 | 1.15±0.17 |
| Fitm1 | 1+0.07 | 1.02+0.12 | 0.93+0.06 | 1+0.12 | 1.68+0.24^a^ | 1.23+0.15 |
| Fitm2 | 1+0.07 | 1.02+0.13 | 0.93+0.06 | 1+0.06 | 1.03+0.11 | 1.03+0.09 |
| **Insulin-like growth factors and binding proteins** | | | | | | |
| Igf1 | 1.00 ± 0.13 | 1.44 ± 0.17^a^ | 1.48±0.12^b^ | 1.00 ± 0.13 | 0.76 ± 0.09 | 1.08±0.17 |
| Igf2 | 1.00 ± 0.08 | 1.75 ± 0.25^a^ | 1.51±0.25^b^ | 1.00 ± 0.14 | 0.83 ± 0.19 | 1.34±0.39 |
| Igfbp1 | 1.00 ± 0.35 | 2.55 ± 0.58^a^ | 2.00±0.4^b^ | 1.00 ± 0.37 | 1.93 ± 0.66 | 1.61±0.76 |
| Igfbp2 | 1.00 ± 0.16 | 2.16 ± 0.32^a^ | 1.47±0.23^b^ | 1.00 ± 0.09 | 1.02 ± 0.10 | 1.05±0.09 |
| Igfbp3 | 1.00 ± 0.19 | 1.15 ± 0.15 | 0.81±0.06 | 1.00 ± 0.14 | 0.94 ± 0.07 | 0.90±0.11 |
|  |  |  |  |  |  |  |

F:WT; M:WT: wildtype offspring of wildtype fathers and wildtype mothers; F:WT; M:eNOS+/-: wildtype offspring of wildtype fathers and eNOS heterozygous mothers; F:eNOS+/-;M:WT: wildtype offspring of eNOS heterozygous fathers and wildtype mothers. Data are given as mean ± SEM. a: p<0.05, F:WT; M:eNOS+/- vs F:WT; M:WT; b: p<0.05, F:eNOS+/-; M:WT vs F:WT; M:WT; c: p<0.05, F:eNOS+/-; M:WT vs F:WT; M:eNOS+/-.

**Supplementary Table 8. Correlation analysis between AUC of plasma glucose and insulin, fat content, glycogen, fumarate, and altered genes in wild type offspring with eNOS+/- mothers.**

| Variable | Male offspring | | | | | Female offspring | | | | |
| --- | --- | --- | --- | --- | --- | --- | --- | --- | --- | --- |
|  | AUC of plasma glucose | AUC of plasma insulin | Fat content | Glycogen | Fumarate | AUC of plasma glucose | AUC of plasma insulin | Fat Content | Glycogen | Fumarate |
| Srebf1c | 0.116 | 0.172 | 0.171 | .684** | 0.041 | 0.131 | 0.018 | -0.308 | 0.375 | 0.407 |
| PPARγ | 0.029 | 0.026 | .511** | -0.15 | .371* | .403** | .447* | 0.065 | -0.171 | -0.018 |
| Gck | -0.264 | 0.064 | -0.313 | 0.211 | -0.203 | -0.222 | -0.319 | -0.351 | -0.161 | 0.032 |
| Pepck (Pck1) | -0.036 | 0.288 | -0.112 | -0.079 | -0.058 | -0.078 | -0.377 | -0.099 | 0.079 | -0.006 |
| Fitm1 | 0.13 | 0.173 | .443** | 0.027 | 0.006 | 0.238 | 0.226 | .508** | 0.247 | -0.264 |
| Igf1 | -0.057 | 0.396 | -.555* | 0.45 | -0.383 | -0.132 | -0.269 | -0.253 | -0.186 | 0.027 |
| Igf2 | 0.028 | 0.333 | -0.319 | 0.442 | 0.089 | -0.209 | -0.425 | -0.348 | 0.06 | -0.014 |
| Igfbp1 | -0.257 | 0.114 | -0.464 | 0.027 | -0.022 | 0.19 | -0.038 | 0.067 | 0.092 | -0.103 |
| Igfbp2 | 0.01 | 0.067 | -.592* | 0.123 | -0.278 | -0.269 | -.681** | -0.202 | -0.343 | -0.153 |

AUC: area under curve. *: p<0.05, **: p<0.01.

**Supplementary Table 9. Correlation analysis between AUC of plasma glucose and insulin, fat content, glycogen, fumarate, and altered genes in wild type offspring with eNOS+/- fathers.**

| Variable | Male offspring | | | | | Female offspring | | | | |
| --- | --- | --- | --- | --- | --- | --- | --- | --- | --- | --- |
|  | AUC of plasma glucose | AUC of plasma insulin | Fat content | Glycogen | Fumarate | AUC of plasma glucose | AUC of plasma insulin | Fat Content | Glycogen | Fumarate |
| Chrebp (Mlxipl) | -0.259 | 0.201 | 0.225 | .487* | -0.116 | 0.28 | .357* | 0.162 | 0.008 | 0.113 |
| PPARα | -.382* | -0.097 | 0.123 | -0.055 | 0.045 | .351* | .411* | 0.12 | -0.045 | -0.236 |
| PPARγ | 0.265 | .723** | .589** | 0.198 | 0.027 | .358* | .423* | 0.158 | -0.011 | -.306* |
| GR (Nr3c1) | -0.089 | 0.201 | -0.187 | 0.481* | -0.469* | -0.096 | 0.25 | 0.084 | -0.194 | -0.236 |
| Tfam | 0.11 | 0.257 | 0.347 | .469* | -0.281 | 0.189 | 0.207 | 0.279 | 0.132 | 0.073 |
| Tfb2m | 0.107 | 0.465* | 0.364 | .611** | -0.277 | 0.222 | 0.265 | 0.252 | 0.021 | 0.032 |
| Nampt | 0.014 | 0.251 | 0.058 | 0.371 | -0.155 | 0.193 | -0.003 | 0.032 | -0.041 | 0.21 |
| Pepck (Pck1) | -0.256 | -0.157 | -0.29 | 0.162 | -0.258 | 0.163 | 0.192 | -0.227 | 0.096 | -0.139 |
| G6Pase (G6pc) | -0.282 | 0.348 | 0.168 | .388* | 0.137 | -0.256 | -0.213 | -0.127 | 0.34 | 0.059 |
| Glut2 (Slc2a2) | 0.124 | 0.126 | 0.129 | .595* | -.701** | -0.248 | 0.085 | 0.332 | -0.391 | -0.204 |
| FAS (Fasn) | 0.307 | 0.179 | 0.146 | 0.316 | 0.1 | -0.009 | 0.016 | .556* | -0.123 | -0.289 |
| Igf1 | 0.014 | 0.345 | -0.039 | 0.053 | -0.319 | -0.211 | -0.214 | -0.172 | -0.312 | -0.159 |
| Igf2 | 0.055 | 0.21 | -0.425 | .600* | -0.147 | 0.001 | -0.178 | -0.336 | -0.241 | -0.208 |
| Igfbp1 | -0.05 | 0.293 | -0.245 | 0.097 | 0.125 | 0.022 | 0.183 | -0.443 | 0.195 | 0.025 |
| Igfbp2 | 0.168 | -0.1 | -0.144 | -0.184 | -0.073 | -0.224 | -0.332 | -0.428 | -0.209 | -0.106 |

AUC: area under curve. *: p<0.05, **: p<0.01.
